# A convenient protocol for establishing a human cell culture model of the outer retina.

**DOI:** 10.12688/f1000research.15409.1

**Published:** 2018-07-18

**Authors:** Savannah A. Lynn, Eloise Keeling, Jennifer M. Dewing, David A. Johnston, Anton Page, Angela J. Cree, David A. Tumbarello, Tracey A. Newman, Andrew J. Lotery, J. Arjuna Ratnayaka

**Affiliations:** 1 Clinical and Experimental Sciences, Faculty of Medicine, MP 806, Tremona Road, University of Southampton, Southampton, Hampshire, SO16 6YD, UK; 2 Biomedical Imaging Unit, MP 806, Tremona Road, University of Southampton, Southampton, Hampshire, SO16 6YD, UK; 3 Biological Sciences, Faculty of Natural & Environmental Sciences, Life Sciences Building 85, University of Southampton, Southampton, Hampshire, SO17 1BJ, UK; 4 Eye Unit, University Hospital Southampton NHS Foundation Trust, Southampton, Hampshire, SO16 6YD, UK

**Keywords:** Retinal Pigment Epithelium (RPE), In-vitro, Cell culture, Disease modelling, Retinopathy

## Abstract

The retinal pigment epithelium (RPE) plays a key role in the pathogenesis of several blinding retinopathies. Alterations to RPE structure and function are reported in Age-related Macular Degeneration, Stargardt and Best disease as well as pattern dystrophies. However, the precise role of RPE cells in disease aetiology remains incompletely understood. Many studies into RPE pathobiology have utilised animal models, which only recapitulate limited disease features. Some studies are also difficult to carry out in animals as the ocular space remains largely inaccessible to powerful microscopes. In contrast, *in-vitro* models provide an attractive alternative to investigating pathogenic RPE changes associated with age and disease. In this article we describe the step-by-step approach required to establish an experimentally versatile *in-vitro* culture model of the outer retina incorporating the RPE monolayer and supportive Bruch’s membrane (BrM). We show that confluent monolayers of the spontaneously arisen human ARPE-19 cell-line cultured under optimal conditions reproduce key features of native RPE. These models can be used to study dynamic, intracellular and extracellular pathogenic changes using the latest developments in microscopy and imaging technology. We also discuss how RPE cells from human foetal and stem-cell derived sources can be incorporated alongside sophisticated BrM substitutes to replicate the aged/diseased outer retina in a dish. The work presented here will enable users to rapidly establish a realistic *in-vitro* model of the outer retina that is amenable to a high degree of experimental manipulation which will also serve as an attractive alternative to using animals. This *in-vitro* model therefore has the benefit of achieving the 3Rs objective of reducing and replacing the use of animals in research. As well as recapitulating salient structural and physiological features of native RPE, other advantages of this model include its simplicity, rapid set-up time and unlimited scope for detailed single-cell resolution and matrix studies.

Research highlights **Scientific benefits:**

- We provide a step-by-step protocol to rapidly establish an *in-vitro* model of the outer retina incorporating the Retinal Pigment Epithelium (RPE) and the supportive Bruch’s membrane.
- We discuss the advantages and limitations of RPE cells (the ARPE-19 cell-line) used in this work.
- This *in-vitro* model allows the use of powerful confocal microscopes (fast, high-resolution imaging) and new platforms such as 3View and Lightsheet.
- Allows a high degree of experimental manipulation.

**3Rs benefits:**

- This *in-vitro* culture model can be used as an alternative to *in-vivo* experiments in spontaneously arising, acutely-induced or transgenic mouse models of retinal degeneration, or be used in parallel with animal studies.
- This model enables users to obtain functional RPE monolayers with desirable physiological and structural features of the native RPE tissue after only 2–4 months in culture.
- Such *in-vitro* RPE monolayers can therefore be used to model disease features which do not manifest in some mouse models for as long as 18 months.

**Practical benefits:**

- This *in-vitro* culture model has a relatively fast set-up period enabling studies after 2–4 months.
- The well-characterised ARPE-19 cell-line used in this work facilitates reproducibility and comparisons with a large body of published literature.
- Cost effective compared to carrying out similar studies *in-vivo*.
- Allows first-line investigations of specific disease pathways *in-vitro*, hence suited to high-throughput drug discovery screens.

**Current applications:**

- This set-up can be used to culture/model primary RPE cells (from porcine, rodent and humans sources), cell-lines such as ARPE-19 as well as human foetal and stem-cell derived RPE from patients.
- Suitable for single-cell level studies and those investigating dynamic changes to the RPE monolayer or the underlying extracellular matrix.
- Used by researchers to study RPE changes in different types of blinding diseases in which the retina irreversibly degenerates including Age-related Macular Degeneration (AMD), Sorsby Fundus Dystrophy and Retinitis Pigmentosa.

***Potential applications:***

- Further refinements to this model can be made by incorporating stem cell-derived RPE directly from patients, synthetic membranes to better mimic the underlying Bruch’s membrane and microfluidic devices to simulate the choroid.
- Sets the standard to recapitulate structural and functional features for future *in-vitro* 3D retinal models.

## Introduction

The retinal pigment epithelium (RPE) consists of a monolayer of largely cuboidal-shaped pigmented cells found beneath the neuroretina and overlying the vascular blood supply of the choriocapillaris. Occupying this strategic position in the outer retina the RPE performs multiple functions which are essential for retinal homeostasis and maintenance of life-long vision. This includes the daily phagocytosis of shed Photoreceptor Outer Segments (POS), re-isomerization of all-trans-retinal to 11-cis-retinal in the visual cycle, protection against effects of photo-oxidation, trans-epithelial transport as well as the polarised secretion of molecules towards the overlying neuroretina and the underlying choroid. The RPE also forms part of the outer blood-retinal barrier (BRB) which functions to confer an immune privileged state within the ocular environment ^1^. Dysfunction or abnormalities of the RPE monolayer is correlated with early stages of pathology linked to a range of ocular conditions such as Age-related Macular Degeneration (AMD), Sorsbys fundus dystrophy, Stargardt disease and Best disease, diabetic retinopathy as well as pattern dystrophies ^1– 3^. However, the origins of RPE dysfunction and how they contribute to such diverse ocular conditions remains incompletely understood.

Numerous *in-vivo* models including non-human primates, pigs, sheep, rabbits and rodents have been used to study retinal pathobiology ^4^. Of these, the most widely used are mice, which show regional differences in Bruch’s membrane (BrM) thickness and photoreceptor density, a similar rod to cone ratio at locations comparable to the peripheral human macula as well as a similar RPE monolayer to humans ^5^. Mice also offer advantages in terms of costs compared to the use of larger animals and the possibility of studying salient disease features in a matter of months. This has led to the use of spontaneously arising ^6^, acutely-induced ^7^ and transgenic mouse models ^8^, or indeed combined models where genetics and diet has been manipulated and mice aged for long periods to bring about disease features ^9, 10^. However, given the lack of anatomical specialisation equivalent to humans, rodent models are of limited value for studies into macular conditions. Moreover, no single mouse model is capable of replicating the full disease spectrum observed in human retinopathies. This has often led to the unnecessary and over-use of poorly characterised rodent models, many of which show only limited disease features and/or have to be aged for long periods before any obvious retinal pathology is detected ^4, 11^. The arrangement of ocular tissues such as the RPE also makes them difficult to image, particularly for studies requiring dynamic, real-time imaging or data at single-cell resolution. Welfare concerns and severity limits of mouse models, other than basic information on animal husbandry, are also poorly reported in the literature. For instance, there is limited data on how a particular genetic alteration or mice maintained over long periods (>18 month) might affect their behaviour and quality of life. In contrast, *in-vitro* models, although simplistic by comparison, are not limited by these issues and boast distinct advantages over mouse models for delineating cellular pathways of damage, or for drug screens to identify effects on a given cell type. Cells cultured under *in-vitro* conditions that recapitulate their *in-situ* environment have been shown to reproduce a phenotype that closely resemble native tissues. These cells not only adopt native-like structural and physiological characteristics, but also a genetic profile closely matching their *in-situ* counterpart. RPE cells were initially grown on plastic substrates and did not exhibit a fully differentiated phenotype. Investigators therefore started culturing RPE monolayers on commercially-sourced transwell inserts with varying pore sizes which mimics important features of the underlying BrM ^2, 12^. The culture of RPE cells on 0.4μm pore-size inserts is now widely regarded to produce the most desirable RPE phenotype ^13^. The presence of a porous underlying substrate allows the RPE to undertake activities such as matrix deposition ^14^ and directional secretion of molecules ^15^ which are key features of these cells. Transwell inserts are now widely used to culture primary porcine ^16^, murine ^17, 18^, human foetal RPE (hfRPE) ^19– 23^ and adult human RPE ^24^ as well as numerous cell-lines including ARPE-19 cells ^18, 19, 25, 26^. Studies have shown that RPE cultured under such conditions display structural and functional characteristics of native RPE cells, albeit to differing extents ^16– 19, 21, 24, 25^. New developments are also incorporated into transwell systems. For instance, recent advances in stem-cell technology, which allows the generation of pluripotent stem-cell derived RPE (PSC-RPE) directly from patients ^27, 28^, are routinely modelled in transwells. This approach has resulted in what many consider to be the current gold-standard in RPE modelling. However, several unresolved issues remain as respective labs use different protocols and appear to differentiate cells to different extents before studies are undertaken. Human PSCs are also limited by effects of senescence and variability between clones. These may cause future difficulties with reproducibility ^29^. RPE cell-lines by contrast, which some may consider to be the poor cousin of PSC-RPE, continue to offer some advantages. The rat immortalised RPE-J cell-line ^30^ and the spontaneously arising human ARPE-19 cell-line ^26^ are two noteworthy examples that have been extensively used in transwell systems. ARPE-19 cells have certain advantages as the RPE-J cells will only proliferate if maintained at 32°C and require retinoic acid for contact inhibition. ARPE-19 cells are also highly characterised, well understood by researchers and widely used for over 2 decades to gain key insights into RPE pathobiology since first described by Dunn and colleagues in 1996 ^26^. The consistency of ARPE-19 cells sourced from commercial suppliers has also contributed to generating comparable and reproducible data across different labs that is as yet unmatched in the field. The culture of ARPE-19 was further optimised in recent years ^25^ such that they exhibit a normal karyotype ^31^, apical-basolateral specialisation ^18^ as well as pigmentation and express characteristic proteins ^18, 25^ including components of the BRB ^18, 25, 32– 34^, polarised secretion ^18, 19, 25^ and phagocytosis ^25, 26, 35^. In fact, new evidence show that when cultured for 4 months in optimised medium, ARPE-19 cells exhibit a comparable transcriptome to the native RPE ^36^. Further refinement in modelling RPE cells on transwell inserts can be anticipated due to advances in new artificial BrM substitutes ^37, 38^. 

A literature search using PubMed Central in May 2018 using the terms ‘Retinal Pigment Epithelium’ and ‘Transwell’, reported a 193% increase of *in-vitro* studies within the past 5 years compared to the previous 5-year period. In contrast, search terms ‘Retinal Pigment Epithelium’ and ‘ *in-vivo*’ revealed a 16% reduction in the number of reports using *in-vivo* models encompassing rodents, rabbits, porcine, bovine and non-human primates over a similar period. Based on these findings and an average annual increase of 4.3% in citations with RPE studies, we estimate that at least 45 publications reporting *in-vivo* work will be replaced by *in-vitro* RPE modelling studies. Given the rapid rate at which RPE modelling work is progressing, this will yield at the very least 188 annual citations by 2023. In this article we provide a detailed step-by-step approach for optimising an *in-vitro* RPE cell model using the spontaneously arisen ARPE-19 cell-line. We also provide steps used to characterise this model, discuss its advantages and limitations as well as how it fulfils the 3Rs objectives.

## Methods

### Establishing the RPE cell model

***Culture of ARPE-19 cells.*** ARPE-19 cells ^26^ were obtained from the American Tissue Culture Collection ( CRL-2302, ATCC, USA) and maintained in a 37°C humidified incubator with 5% CO _2 _atmosphere and 95% air. Cells were cultured in an optimised medium comprising Dulbecco’s modified Eagle’s Medium (DMEM) with 4.5 g/l L-D glucose (high glucose), L-glutamine and pyruvate (41966–029, Life Technologies, UK) supplemented with 1% heat inactivated foetal calf serum (N4762, Sigma Aldrich, UK) and 1% of the penicillin streptomycin stock solution (10,000 units/ml penicillin, 10mg/ml streptomycin in 0.85% saline; P4333, Sigma Aldrich, UK) ^25^. Cells cultured in T25cm ^2^ flasks were maintained in a 5ml volume with a complete media change performed every 2–3 days. Cells cultured on Corning® 12mm, 0.4μm pore, PET Transwell® Permeable Supports (CLS3460, Sigma Aldrich, UK) were maintained in 0.5ml and 2ml of volume of media in apical and basal chambers. Cells grown on Corning® 24mm, 0.4μm pore, PET Transwell® Permeable Supports (CLS3450, Sigma Aldrich, UK) were maintained in 1.5ml and 3ml volume of media in apical and basal chambers. A complete media change in the apical chamber and a 20% (v) change in the basal compartment was performed every 2–3 days. Cells were used between passages 23–27. 

***Fibronectin coating of transwell inserts.*** Lyophilised fibronectin (F2006, Sigma Aldrich, UK) was prepared to a final concentration of 50μg/ml in double distilled water (ddH _2_0), and applied to the apical surface of transwell inserts. A volume of 0.25ml or 0.6ml was used for 12mm and 24mm inserts respectively. Transwells were partially covered in a laminar flow hood and allowed to dry overnight, after which any residual fibronectin was aspirated and inserts washed in 1x phosphate buffered saline (PBS, Sigma Aldrich, UK).

***Passage of ARPE-19 cells.*** ARPE-19 cells were grown in T25cm ^2^ flasks for up to 3 weeks prior to passaging at a 1:3 ratio. This was achieved by washing cells in Ca ^2+^ and Mg ^2+^ free Hank’s Balanced Salt Solution (HBSS; 14065049, Life Technologies, UK) and incubation with 1.5ml 0.25% Trypsin/EDTA (25200056, Life Technologies, UK) for 6 minutes, followed by neutralisation/trituration with 7ml volume of complete medium. The cell suspension was centrifuged at 125g for 5 minutes after which the pellet was suspended in fresh medium. Cells were seeded on fibronectin coated/uncoated 0.4μm PET transwell inserts (Corning, UK) at a density of 1.25×10 ^4^ and 5×10 ^4^ for 12mm or 24mm inserts, and left undisturbed for 4 days to facilitate cell adhesion prior to media change. The culture media used in apical and basal transwell compartments were identical. Long-term cultures were maintained for 2–4 months before assessing characteristic structural RPE features or expression of cell-specific and barrier proteins. Functional assays were carried out in 4 month old cultures.

### Characterisation and validation of the RPE cell model

***Confocal Immunofluorescence microscopy.*** ARPE-19 cultures were rinsed in 1xHBSS, fixed in ice-cold PBS containing 4% formaldehyde for 30 minutes at 4°C and washed three times in 1x PBS. Blocking and permeabilisation were achieved by incubation with 5% normal goat serum (NGS; G9023, Sigma Aldrich, UK) in 1x Phosphate Buffered Saline with Triton X-100 (PBST, Sigma Aldrich, UK) for one hour prior to the addition of primary antibody (prepared in blocking buffer) which was incubated overnight at 4°C. The following primary antibodies were used: ZO-1 (1:100, RRID: AB_2533456, Thermo Fisher Scientific, UK), Occludin (1:100, RRID: AB_2533977, Thermo Fisher Scientific, UK), RPE65 (1:100, RRID: AB_2181006, Abcam, UK) and alpha 1 Na ^+^/K ^+^ ATPase (1:100, RRID:AB_306023, Abcam, UK). The following day, cells were washed in 0.05% PBST and incubated with the appropriate Alexa Fluor® labelled secondary antibody (RRID: AB_2534115, RRID: AB_2534085, RRID: AB_2534116, RRID: AB_2534087, RRID: AB_2534114, RRID: AB_2534064, Life technologies, UK) at a dilution of 1:200 prepared in 0.05% PBST for 1 hour. This was followed by three washes in 1× PBS and one wash with ddH _2_0 after which cells were incubated with 1μg/ml 4’, 6’-diamino-2-phenylindole (DAPI; D9542, Sigma Aldrich, UK) for 10 minutes. Inserts were washed an additional three times in ddH _2_O and mounted between two glass coverslips with Mowiol® mounting medium (Harco Chemical Company Ltd., UK) prior to imaging with a Leica SP5 or SP8 laser-scanning confocal microscope (Leica Microsystems, UK).

***Transmission electron microscopy.*** Transwell inserts were processed for transmission electron microscopy (TEM) by first washing in 1X HBSS, followed by immersion in primary fixative (3% glutaraldehyde and 4% formaldehyde in 0.1M PIPES [Agar Scientific, UK]; pH 7.2) for 1 hour, after which they were rinsed twice for 10 minutes in 0.1M PIPES before post fixation for 1 hour with 1% osmium tetroxide in 0.1M PIPES. Inserts were rinsed twice in 0.1M PIPES and once in ddH _2_O, after which they were stained in 2% (aqueous) uranyl acetate for 20 minutes. Samples were subsequently dehydrated by passing the inserts through a series of ethanol gradients (30%, 50%, 70% and 95%) for 10 minutes each, followed by two 20 minute incubations in absolute ethanol (100%). A link reagent acetonitrile (Agar Scientific, UK) was then applied to inserts for 10 minutes, after which samples were incubated overnight with at a 1:1 ratio of acetonitrile to Spurr resin (Agar Scientific, UK). The following day, cells were incubated with Spurr resin for 6 hours and embedded and polymerised in fresh resin at 60°C for 24 hours. Ultrathin sections were cut using a Reichert Ultracut E (Leica Microsystems, UK), collected on 200 mesh carbon and formvar coated copper grids and stained with Reynolds Lead Stain (Agar Scientific, UK). Cross sections of cultures on transwell inserts were visualised using a Hitachi 7000 transmission electron microscope (Hitachi, Germany) fitted with a SIS Megaview III camera (EMSIS, Germany).

***Trans-epithelial Electrical Resistance measurements.*** Trans-epithelial Electrical Resistance (TEER) were carried out over a 3 month period using an EVOM ^2^ epithelial voltohmmeter and 4mm STX2 chopstick electrode (EVOM ^2^; 300523, World Precision Instruments Inc., USA). Briefly, the electrode was sterilised in 70% ethanol, rinsed in ddH _2_O and equilibrated in pre-warmed culture medium, before being simultaneously introduced into the apical and basal transwell compartments. Measurements were recorded from at least three separate wells per experiment. In each case, five measurements were recorded per well to obtain an average value. The reference value from a fibronectin-coated insert and devoid of cells was subtracted from the average value to yield a net TEER measurement. This was subsequently corrected for the growth area using the following formula ( Equation 1). Measurements were performed at room temperature within 6 minutes of removing cultures from the incubator. A full media change was also performed after weekly measurements to minimise the risk of contamination.

*Final TEER (Ω/cm ^2^) = Net TEER (Ω) × Area of Transwell insert (cm ^2^)*            Equation 1

***ELISA studies.*** Secreted levels of human vascular endothelial growth factor (VEGF: isoforms 165, 121) and pigment epithelium derived factor (PEDF) in apical and basal transwell compartments were quantified using the Novex® human VEGF (KHG0111, Life Technologies, UK) and human PEDF (RD191114200R, BioVendor, Germany) solid-phase sandwich ELISA. Conditioned media was collected from 2 month old ARPE-19 cultures (n=3) and diluted 1:1 and 1:9 prior to VEGF and PEDF ELISAs, respectively. Experiments were carried out in triplicate and followed the manufacturers’ instructions. Optical densities were determined by measuring the absorbance at 450nm using a micro-titre plate reader (FLUOstar Optima; BMG LABTECH, UK) and accounting for the 570nm wavelength correction.

***Preparation of POS-FITC.*** Porcine eyes (maximum of 2 days post mortem) were sourced from a butcher. An incision was made proximate to the ora serata after which the anterior ocular portion was removed and retinae detached gently from the underlying RPE. These were subsequently pooled in KCl buffer (0.3M KCl, 10mM HEPES, 0.5mM CaCl _2_, 1mM MgCl _2_; pH 7.0) with 48% sucrose (w/v), agitated for 2 minutes and centrifuged at 5000g for 5 minutes to facilitate POS detachment. The resultant supernatant containing isolated POS was filtered through a sterile gauze into 1.5ml Eppendorf tube containing an equal volume of KCl buffer without sucrose. POS were pelleted by centrifugation at 4,000g for 7 minutes, washed three times in 1× PBS and re-suspended in DMEM with 2.5% sucrose (w/v). POS were covalently tagged to Fluorescein isothiocyanate (FITC) by incubation with 5ml labelling buffer (20Mm phosphate buffer pH 7.2, 5mM taurine with 10% w/v sucrose) and 1.5ml FITC stock solution (2mg/mL FITC isomer I in 0.1M Na _2_CO _3_ buffer; pH 9.5) at room temperature for 1 hour in the dark on a Stuart SB2 Rotator (Camlam Ltd, UK). POS-FITC conjugates were pelleted by centrifugation at 3,000g for 5 minutes and re-suspended in DMEM with 2.5% sucrose (w/v). Isolated POS can be stored for up to 6 months at -80°C. The total protein content in preparations was quantified using a BCA assay (23225, Thermo Fisher, UK) following the manufacturer’s instructions.

***POS feeding assay and assessment of trafficking dynamics.*** ARPE-19 monolayers on transwell inserts were incubated at 17°C for 30 minutes prior to exposure with 4mg/cm ^2^ POS-FITC for a further 30 minutes at 17°C. This facilitates maximal POS binding with minimal internalisation ^39^ to initiate a pulse-chase assay. The POS-FITC solution was aspirated to remove unbound POS. Cultures were supplemented with fresh media and returned to a humidified 37°C incubator with 5% CO _2_ and 95% air. Cells were subsequently fixed at 2, 4, 6, 12, 24 and 48 hours with 1×PBS containing 4% formaldehyde for 30 minutes at 4°C, after which they were incubated with 1% BSA in PBS-Tween to block/permeabilise cells for 30 minutes. Cultures were probed overnight at 4°C with the following primary antibodies prepared in blocking buffer. Rab 5 (1:200, RRID: AB_470264, Abcam, UK), Rab 7 (1:200, RRID: AB_2629474, Abcam, UK), LAMP1 (1:1000, RRID: AB_775978, Abcam, UK), LAMP2A (1:1000, RRID: AB_775981, Abcam, UK) and LC3B (1:200, RRID: AB_881433, Abcam, UK). Excess antibodies were washed three times in 1xPBS, following which cultures were incubated with the appropriate Alexa Fluor® secondary antibody (RRID: AB_2534115, RRID: AB_2534085, RRID: AB_2534116, RRID: AB_2534087, RRID: AB_2534114, RRID: AB_2534064, Life technologies, UK) for one hour at room temperature. Inserts were finally washed three times in 1×PBS, once in ddH _2_O and counterstained with 1μg/ml DAPI for 10 minutes. Samples were mounted between two glass coverslips with Mowiol® mounting medium and imaged using an SP8 laser-scanning confocal microscope (Leica Microsystems, UK). Quantification of POS-FITC co-localisation with various endocytic/phagocytic, lysosomal and autophagy compartments (n=10 cells/compartment/time point) was performed using Volocity software, version 6.1.1 (Perkin Elmer, UK), which employs the Costes *et al*. automated statistical algorithm ^40^. Co-localisation values were plotted for each compartment as a function of time.

***Statistical Analysis.*** Statistical analyses were conducted using the GraphPad Prism 7 Software (GraphPad, US). Values were first assessed to ensure data met assumptions of the selected statistical test. Tests for each experiment appear in figure legends. Briefly, ELISA quantification was assessed using the unpaired student’s t-test, whilst TEER were evaluated using a one way ANOVA and Tukey’s multiple comparisons tests. In both cases, a single well corresponded to an experimental unit. Data is presented as means ± standard error of the mean (SEM) where n represents independent experiments. Statistical significance is denoted as * p ≤ 0.05, ** p ≤ 0.01, *** p ≤ 0.001 and **** p ≤ 0.0001.

## Protocol

**Figure 1.**
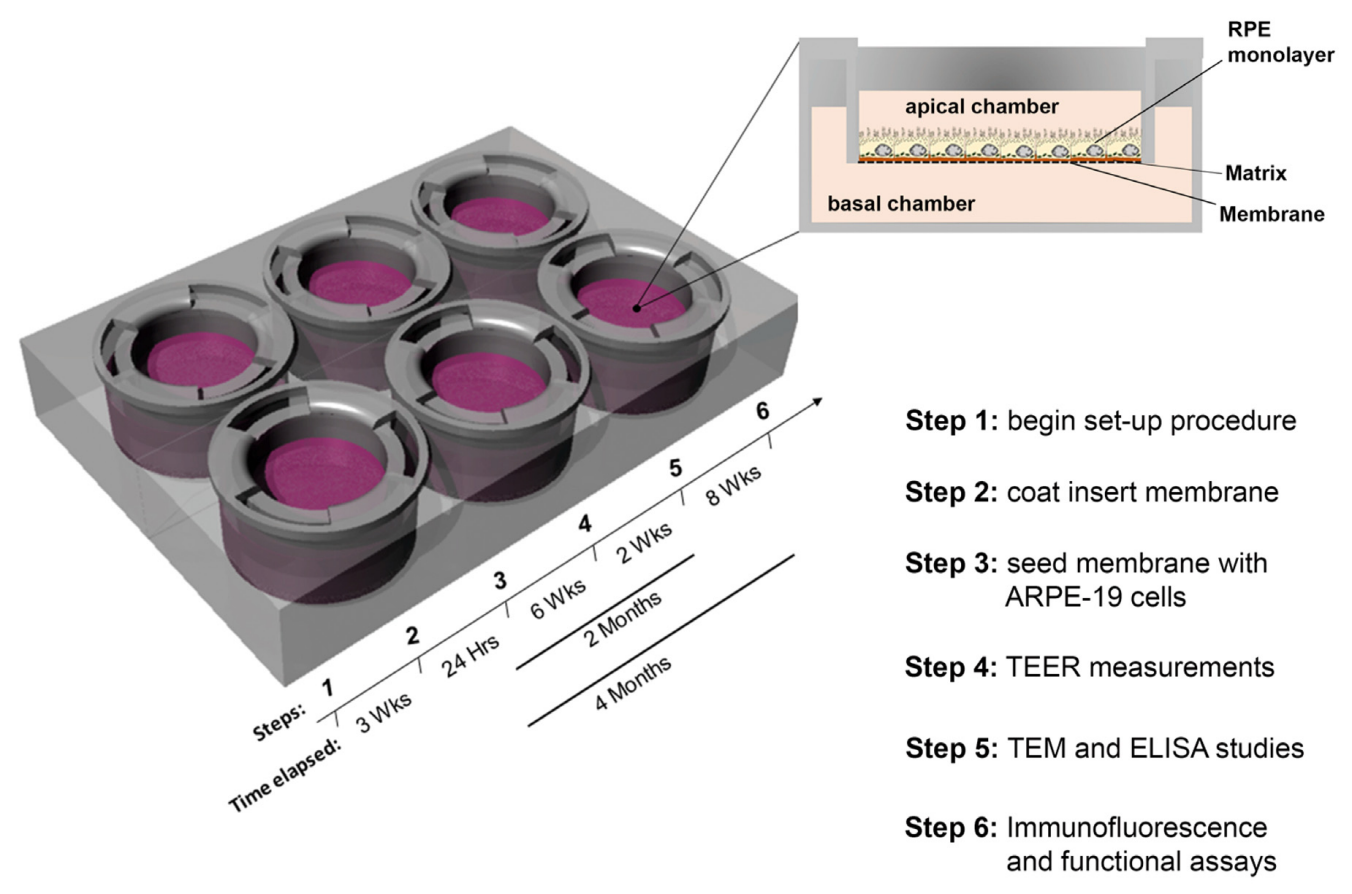
Schematic of transwell plate and cross-section showing Retinal Pigment Epithelial (RPE) cells on porous membrane within insert. Key points in the establishing and validating RPE cultures on transwells. The steps and time periods required for this procedure are indicated alongside each outcome measure.

Here we describe the step-by-step procedure required for establishing and validating long-term cultures of ARPE-19 monolayers on transwell inserts. A schematic highlighting the sequence of steps and timelines are summarised in Figure 1.

### Protocol for establishing the culture model

**Step 1:** Preparation of cell culture media

ARPE-19 cells (CRL-2302™ ATCC®, USA) require growth in optimised culture media, which can be prepared according to Table 1. Freshly prepared cell culture media is passed through a vacuum filter to maximise sterility and used within 2 weeks. A volume of 250ml is sufficient for the culture of 6 transwell plates of 12mm diameter inserts or 4 plates of 24mm diameter inserts ( Table 4) for a period of approximately 2 weeks. Volumes should be scaled according to the size and number of desired transwells as storage of media for longer periods is not recommended.

**Table 1.** Materials used to prepare optimised ARPE-19 culture medium.

Product	Product Reference	Supplier	Volume (ml)	Storage
DMEM, high glucose, pyruvate	41966-029	Life Technologies, UK	245	4°C
Heat inactivated new born calf serum (FCS)	N4762	Sigma Aldrich, UK	2.5	Short term: 4°C Long term: -20°C Note: Avoid freeze-thaw of FCS by preparing aliquots.
Penicillin-Streptomycin	P4333	Sigma Aldrich, UK	2.5	-20°C

Abbreviations: Dulbecco’s Modified Eagle Medium (DMEM)

**Step 2:** Culture of ARPE-19 cells

ARPE-19 cells should be maintained in a humidified incubator set at 37°C with an atmosphere of 5% CO _2_. Cells should be cultured in a T25cm ^2^ flask containing 5ml of freshly prepared media and passaged at a 1:3 ratio when confluent or approximately every 3 weeks. A complete media change should be performed every 2–3 days to retain physiological glucose levels ^41^. Although it is possible to maintain ARPE-19 cells in T25cm ^2^ flasks for longer periods, cells becoming increasingly difficult to passage following the formation of an underlying extracellular matrix. Passaging involves removal of conditioned medium and washing cells with 1xHBSS followed by exposure to 1.5ml 0.25% trypsin/EDTA for 6 minutes in an incubator. The trypsin/EDTA solution is neutralised using 7ml of complete media and triturated to obtain single cells after which the resulting suspension is centrifuged at 300g for 5 minutes. The pellet is re-suspended in a volume of freshly prepared culture media and split at a 1:3 ratio between T25cm ^2^ flasks. The importance of correct cell passaging to maintaining an epithelial phenotype is discussed elsewhere ^42^. For the long term storage in liquid nitrogen (-195.8°C) cells should be suspended in the desired concentration in freezing medium comprising 75% complete culture medium and 25% dimethylsulfoxide (DMSO; S-002-M, Sigma Aldrich, UK). For ARPE-19 cells we recommend 1ml aliquots of 1x10 ^6^ cells. These must first be frozen in a suitable container (Mr Frosty™, Thermo Fisher Scientific UK) or equivalent with 100% isopropanol at -80°C overnight prior to storage in liquid nitrogen.

**Step 3:** Coating transwell inserts with fibronectin

**Figure 2.**
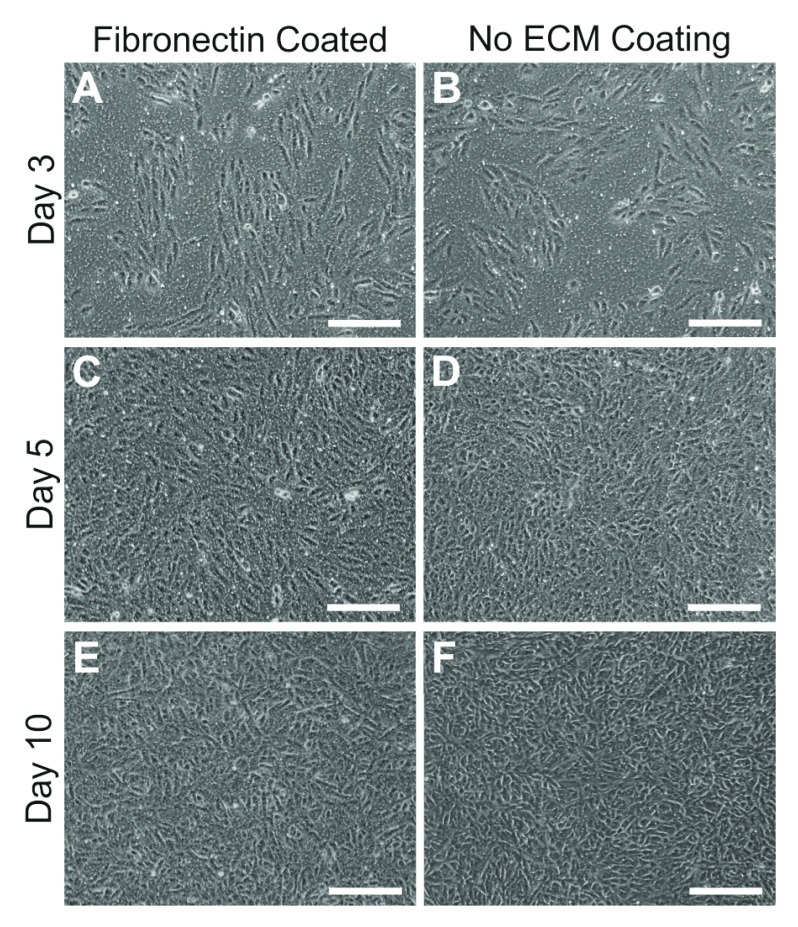
Attachment and growth of ARPE-19 cells with or without an underlying fibronectin matrix. We tested effects of an extracellular matrix (ECM) such as fibronectin on the ability of cells to form a monolayer on transwell membranes. Inserts coated with fibronectin [ **A**, **C**, **E**] or those without any coating [ **B**, **D**, **F**] were imaged over several days after seeding. No differences were observed in cell attachment or growth. Contact inhibition proceeded cell differentiation after approximately 10 days in culture. Scale bars correspond to 200μm.

Prepare lyophilised fibronectin (F2006, Sigma Aldrich, UK) to a final concentration of 50μg/ml by adding 20ml of sterile ddH _2_O. We recommend preparing an initial 5ml solution using sterile ddH _2_O followed by transfer to a 50ml falcon containing 15ml sterile ddH _2_O. The fibronectin solution should be used immediately and is sufficient to coat six 12mm diameter or five 24mm diameter transwell plates, or should be divided into aliquots for storage at -20°C. Users should apply the stock solution to the apical transwell compartment as indicated in Table 2. Ensure that the entire surface of the membrane is covered after which transwells are left partially covered in a laminar flow hood overnight. The following day any residual fibronectin should be aspirated and wells washed with 1x sterile PBS for cell seeding ( Table 3). We observed that ARPE-19 cells readily proliferate and mature to form *in-situ* RPE-like monolayers on an underlying fibronectin matrix although others had used a laminin substrate ^26^. Consequently, work presented here are carried out on transwells coated with fibronectin. It is also possible to culture cells in the absence of an underlying coating on transwell membranes ( Figure 2). ARPE-19 cultured without an extracellular matrix (ECM) substrate develop pigmentation, establish a trans-epithelial barrier and secrete proteins directionally ^25^. The culture of cells without an underlying coating is particularly useful for studies in which *de-novo* synthesis/deposition of extra cellular components and their turnover can be monitored without any influence of artificial substrates.

**Table 2.** Coating volumes for extracellular matrix proteins such as fibronectin used in transwell inserts of different diameters.

Culture surface	Growth Surface Area (cm ^2^)	Coating Volume (ml)	Wash Volume (μl)
Corning ^®^ 24mm, 0.4μm pore, PET Transwell ^®^ Permeable Support	4.67	0.6	1
Corning ^®^ 12mm, 0.4μm pore, PET Transwell ^®^ Permeable Support	1.12	0.25	0.4

Abbreviations: Polyethylene terephthalate (PET)

**Table 3.** Cell seeding densities. Seeding densities used to start ARPE-19 cultures on transwell inserts of different diameters.

Transwell size	Seeding density (cells/well)
Corning ^®^ 24mm, 0.4μm pore, PET Transwell ^®^ Permeable Support	5×10 ^4^
Corning ^®^ 12mm, 0.4μm pore, PET Transwell ^®^ Permeable Support	1.25×10 ^4^

Abbreviations: Polyethylene terephthalate (PET)

**Table 4.** Media volumes and feeding regimes used for the culture of ARPE-19 cells in transwell inserts. Media changed every 2–3 days.

Transwell size	Volume in each transwell compartment (ml)	Volume change (%)
Corning ^®^ 24mm, 0.4μm pore, PET Transwell ^®^ Permeable Support	Upper chamber: 2 Lower chamber: 3	Upper chamber: 100 Lower chamber: 20
Corning ^®^ 12mm, 0.4μm pore, PET Transwell ^®^ Permeable Support	Upper chamber: 0.5 Lower chamber: 1.5	Upper chamber: 100 Lower chamber: 20

Abbreviations: Polyethylene terephthalate (PET)

Technical tip: Rapid thawing of soluble fibronectin can result in the irreversible precipitation of proteins. To avoid this we suggest that the stock solution should be gradually thawed at 4°C.

**Step 4:** Seeding and culture of ARPE-19 cells on transwell inserts

Cells are passaged as described in step 2. A confluent flask of T25cm ^2^ ARPE-19 cells yield between 3–5 million cells. Consequently, one T25cm ^2^ flask is sufficient to seed 10 plates of 24mm diameter transwell inserts or 20 plates of 12mm diameter inserts. Cells are seeded on fibronectin coated transwells ( Table 3), although the benefit of using uncoated transwell membranes have also been discussed. Culture media are applied to the apical and basal transwell compartment at least one hour prior to cell seeding ( Table 4). Following seeding, we recommend leaving cultures undisturbed for approximately 4 days prior to the first media change. Cultures are maintained in a 37°C incubator with an atmosphere of 5% CO _2_ and media changed every 2–3 days ( Table 4). After approximately 1 week cultures appear confluent and by 2 weeks exhibit characteristic cobblestone morphology. Obvious signs of pigmentation will develop after 3–4 months, although some evidence of pigmentation is apparent under light microscopy after 2 months. The size of pores in transwell membranes are known to influence RPE morphology ^13^ hence we suggest users adhere to these recommendations. Cultures are maintained for a minimum of 2 months prior to validation studies. For functional experiments, such as POS feeding assays, we recommend maintaining cultures for approximately 4 months.

Technical tip: Pipette solutions along plastic walls of the transwell chamber at a steady state during media changes to avoid inducing cell stress or cell detachment. When removing media we recommend first tilting the transwell plate to a 45° angle to avoid disturbing the RPE monolayer.

### Protocol for characterising and validating the culture model

In this section we describe the steps used to validate and characterise ARPE-19 monolayers on transwell inserts. These approaches however may be adopted for the culture of RPE from different sources, although the time taken to obtain monolayers displaying physiological and structural features akin to the native RPE may vary depending on the specific type/source of RPE cells.

**Step 5:** Confocal immunofluorescence studies of ARPE-19 monolayers

**Figure 3.**
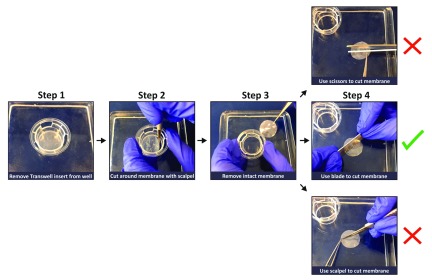
A convenient procedure for removing and sectioning membrane inserts from transwells. Transwell membranes can be detached by making incisions with a scalpel following the circumference of the plastic chamber. The insert lip may be used as an indicator of the boundary. Once removed, the detached membrane can be guillotined using a sharp razorblade. We advise against slicing or cutting using sheer forces as this is likely to damage or dislodge the Retinal Pigment Epithelial monolayer.

**Figure 4.**
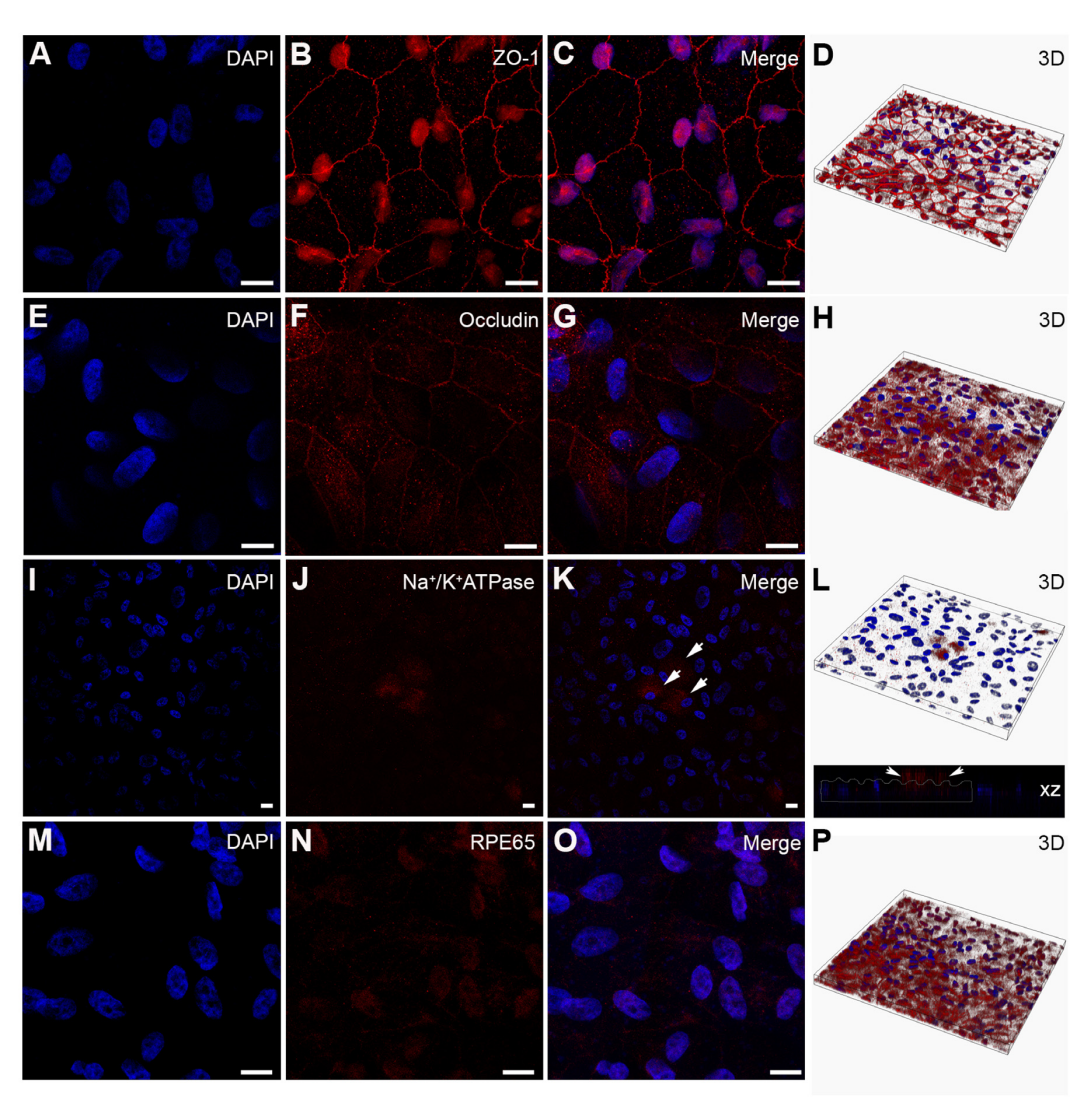
Characterisation of ARPE-19 monolayers cultured on transwell inserts. Cultures were probed for [ **A**– **D**] the early tight junctional marker Zonula Occludens-1, which showed cobblestone morphology characteristic of Retinal Pigment Epithelial (RPE) cells. [ **E**– **H**] We also observed expression of the mid-late barrier protein occludin, although their staining was somewhat weaker. [ **I**– **L**] The epithelial transporter Na ^+^/K ^+^ ATPase was observed in APPE-19 monolayers (arrows) but was not evident in all cells. Staining was however observed predominantly on the apical RPE surface, a feature reported in highly differentiated RPE cells. [ **M**– **P**] The cell-specific marker RPE-specific 65 kDa protein (RPE65) was also observed after 2 months in culture. Nuclei were counterstained with DAPI (blue). [ **A**– **C**, **E**– **G**, **I**– **K**, **M**– **O**] show representative en-face confocal images whilst [ **D**, **H**, **L**, **P**] show corresponding z-plane reconstructions. Scale bars correspond to 20μm.

Cells that have been in culture for at least 2 month are used to ensure RPE monolayers had adopted structural and physiological features of native RPE. Transwell inserts are washed with 1x sterile PBS prior to fixation in 4% PFA for 30 minutes. Each transwell membrane is removed from its insert by running a blade along the circumferential ring ( Figure 3). The amount of material (RPE monolayers) required to carry out experiments may be maximised by sectioning transwell membranes into multiple sections, although caution must be exercised to prevent disturbing the delicate cell layer. We recommend using a sharp razor blade to guillotine sections of the membrane outright as cutting or slicing generates sheer forces which rucks membranes leading to cell detachment ( Figure 3). Transwell membranes are washed three times in 1x PBS and blocked/permeabilised in 5% NGS in 0.1% PBST for 1 hour. A battery of antibodies are used to probe for components of the BRB (ZO-1 and Occludin), to assess RPE polarisation (Na ^+^/K ^+^ ATPase) and to detect expression of the cell-specific marker (RPE65), although other proteins such as CRALBP may also be included. Readers are also referred to studies described in Ahmado *et al.*, 2011 ^25^. Primary Antibodies are diluted 1:100 in blocking buffer for incubation at 4°C overnight ( Table 5). The following day membranes are washed three times in 0.05% PBST followed by incubation with the appropriate Alexa Fluor® labelled secondary antibody (RRID: AB_2534115, RRID: AB_2534085, RRID: AB_2534116, RRID: AB_2534087, RRID: AB_2534114, RRID: AB_2534064, Life Technologies, UK) prepared in 0.05% PBST for 1 hour. Membranes are washed three times in ddH _2_O to remove any unbound secondary antibody and mounted between two glass coverslips with Mowiol® mounting medium (Harco Chemical Company Ltd., UK). We recommend sandwiching membranes between 2 glass coverslips as opposed to pairing a single coverslip with a thicker glass slide as this allows either side of the sample to be imaged without potential optical interference from the porous membrane. Z-stack image are captured with a laser-scanning confocal microscope ( Supplementary Figure S1 and Figure 4). We recommend a minimum optical slice thickness of 1μm through z-stacks to help assess the polarised expression of RPE markers.

**Table 5.** List of primary antibodies used for confocal immunofluorescence studies.

Primary antibody	Supplier	Product number	RRID	Host species	Clone/isotype	Dilution
Zonula Occludens-1 (ZO-1)	Thermo Fisher Scientific	40-2200	AB_2533456	Rabbit	pAb/IgG	1:100
Occludin	Thermo Fisher Scientific	71-1500	AB_2533977	Rabbit	pAb/IgG	1:100
Retinal Pigment Epithelium- specific 65 kDa protein (RPE65)	Abcam	ab78036	AB_2181006	Mouse	mAb, IgG1	1:100
alpha 1 Na ^+^/K ^+^ ATPase	Abcam	ab7671	AB_306023	Mouse	mAb, IgG1 Kappa	1:100
Rab 5	Abcam	ab18211	AB_470264	Rabbit	pAb/IgG	1:200
Rab 7	Abcam	ab137029	AB_2629474	Rabbit	mAb/IgG	1:200
LAMP1	Abcam	ab24170	AB_775978	Rabbit	pAb/IgG	1:1000
LAMP2A	Abcam	ab18528	AB_775981	Rabbit	pAb, IgG	1:1000
LC3B	Abcam	ab48394	AB_881433	Rabbit	pAb/IgG	1:200

Technical tip: Pores within the membrane may be used as a reference point to help orient the position of apical and basolateral RPE surfaces. However, users may have to account for small undulations in the membrane which will alter the focal plane across the sample.

**Step 6:** Transmission electron microscopy studies of ARPE-19 cultures

**Figure 5.**
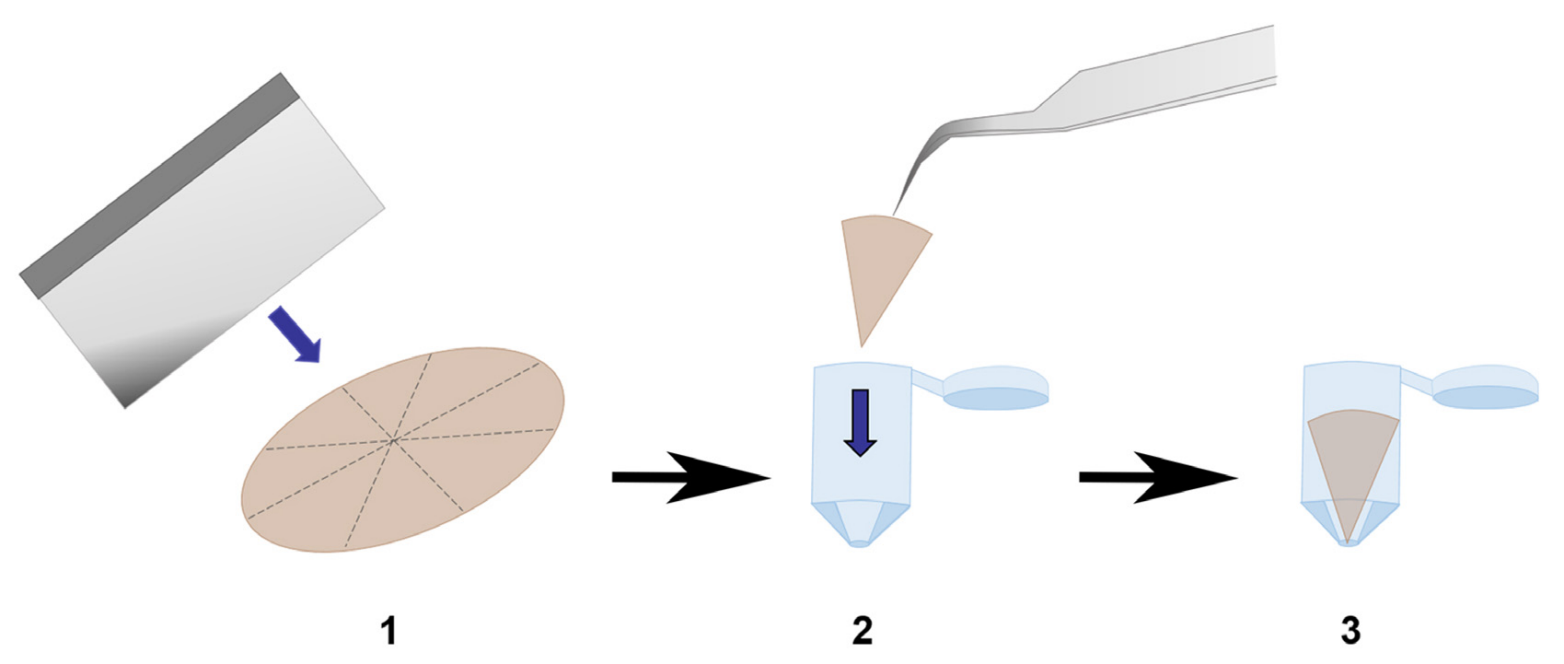
Preparation of polyethylene terephthalate (PET) membranes for transmission electron microscopy (TEM). Schematic outlining steps carried out to embed segmented transwell membranes into capsules containing fresh Spurr resin. The apex is positioned downwards, which greatly assists with cutting sections for TEM.

**Figure 6.**
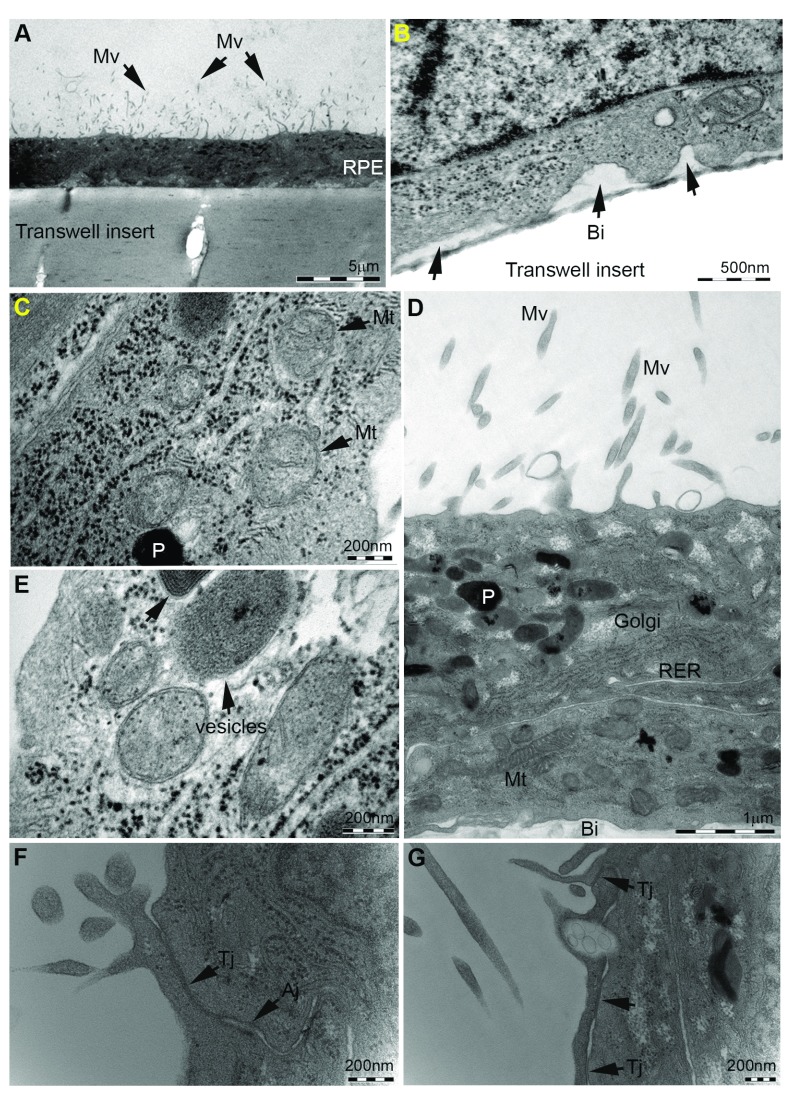
Ultrastructural studies of ARPE-19 monolayers cultured on transwell inserts. Cultures were assessed by transmission electron microscopy to determine the extent of Retinal Pigment Epithelium (RPE) structural specialisation on transwell inserts. [ **A**] Cross-section of the RPE monolayer showing microvilli (Mv) on apical surface (arrows). Pores within the polyethylene terephthalate (PET) membrane are also visible. Scale bar corresponds to 5μm. [ **B**] Basolateral infolds (Bi) in RPE cells adjacent to the transwell membrane (arrows) are evident, under which we have previously observed the accumulation of sub-RPE deposits in long-term culture ^18^. Scale bar corresponds to 500nm. [ **C**– **E**] Intracellular organelles including mitochondria (Mt), vesicular compartments such as phagosomes/endosomes and lysosomes, rough endoplasmic reticulum (RER), Golgi and pigment molecules (P) were evident, indicating apical-basolateral specialisation recapitulating arrangement of *in-situ* RPE. Scale bars correspond to 200nm in [ **C**, **D**] and 1μm in [ **D**]. [ **F**– **G**] We also observed the presence of tight Junctions (Tj) and adherens Junctions (Aj) in apical borders of RPE cells (arrows). Scale bars correspond to 200nm. TEM micrograph in panel **B** was published previously ^18^ under the Creative Commons licence.

Transmission Electron Microscopy (TEM) studies are performed on monolayers cultured for at least 2 months to ensure structural specialisation of the apical and basolateral RPE surfaces. Inserts are washed in 1x HBSS immediately following removal of conditioned media to prevent dehydration and fixed in primary fixative (3% glutaraldehyde and 4% formaldehyde in 0.1M PIPES; pH 7.2) for one hour. Samples are subsequently washed twice in 0.1M PIPES and post fixed in 1% buffered osmium tetroxide (Oxkem, UK) prepared in 0.1M PIPES for 1 hour. Following fixation, samples are rinsed twice in 0.1M PIPES, once in ddH _2_0 and stained with 2% (aqueous) uranyl acetate (Agar Scientific, UK) for 20 minutes. At this stage membranes may be submerged in a 30% ethanol solution for removal from transwells. It is vital that this process is carried under an appropriate liquid (buffer, ethanol) to prevent the cells drying out which would render them useless for microscopy. Samples are passed successively through a graded series of ethanol concentrations (30%, 50%, 70% and 95% ethanol) for 10 minutes each, followed by two successive incubation periods in absolute ethanol for 20 minutes to achieve optimal dehydration. The link reagent acetonitrile (Fisher Scientific, UK) is applied for 10 minutes and membranes incubated in a mixture containing an equal ratio of acetonitrile to Spurr resin overnight. In our experience, Spurr resin appears to be the best medium to effectively bond filters, although sections often split along the resin/filter interface during sectioning and when viewing under the microscope. The following day, samples are incubated for an additional 6 hours in Spurr resin and embed in fresh Spurr resin for polymerisation at 60°C for 24 hours. Samples should be embedded in Spurr resin as triangular slices with the apex of the triangle positioned towards the bottom of the embedding capsule ( Figure 5), which facilitates ease of cutting. This procedure is carried out without a rotator as cells could otherwise detach from the underlying membrane. Ultrathin/silver TEM sections are prepared using a Reichert Ultracut E ultramicrotome and collected on 200 mesh carbon and formvar coated copper palladium grids. We advise against the use of chloroform to stretch sections which exacerbates potential separation of the resin/membrane interface during sectioning. Sections are subsequently stained with Reynold’s lead stain and visualised using a Hitachi 7000 transmission electron microscope fitted with a SIS Megaview III plate EMSIS camera ( Figure 6).

Technical tip: We recommend viewing samples starting at a lower magnification with the electron beam spread widely in order to minimise shrinkage or movement across sections, and to protect against the possibility of splitting at the resin/membrane interface.

**Step 7:** Trans-epithelial electrical resistance measurement of ARPE-19 cultures

**Figure 7.**
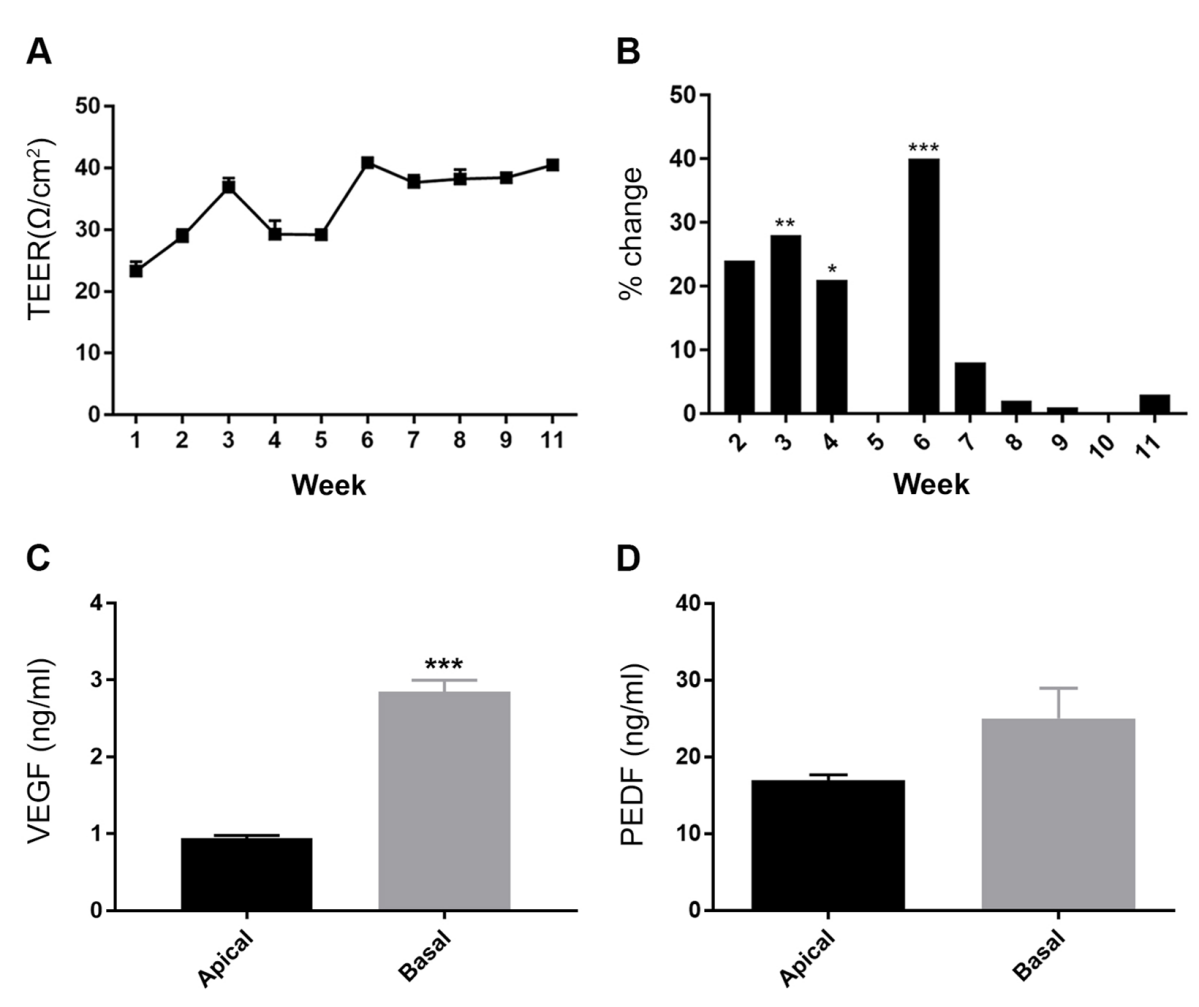
Physiological studies of ARPE-19 monolayers cultured on transwell inserts. Trans-epithelial Electrical Resistance (TEER) measurements were obtained from long-term cultures to evaluate effectiveness of the Retinal Pigment Epithelial barrier. [ **A**] Measurements were conducted from transwells (n=3) at weekly time intervals after seeding. Values were plotted as a percentage change from the previous measurement which show a gradual increase as junctions form and mature. [ **B**] Fluctuations between average weekly TEER were observed prior to week 6 (p=0.009 at 3 weeks, p=0.013 at 4 weeks and p=0.001 at 6 weeks, one-way ANOVA with Tukey’s multiple comparisons) after which a stable value of 40.72 Ω.cm ^2^ was achieved. Data is presented as mean ± SEM. Next, we quantified polarised secretion of Vascular Endothelial Growth Factor (VEGF) and Pigment Epithelium Derived Factor (PEDF) by ARPE-19 cells. Conditioned media was collected (n=3) after 72 hours and proteins quantified by ELISA. [ **C**] The apical compartment was found to contain 0.942 ± 0.035ng/ml of VEGF compared to 2.852 ± 0.145ng/ml in the basal chamber, which was statistically significant (p=0.0002). [ **D**] PEDF concentrations in the apical compartment was 16.95 ± 0.72ng/ml compared to 25.05 ± 3.93ng/ml in the basal chamber. There were no significant differences (p= 0.112) although more PEDF was secreted via the basolateral RPE surface. Data is presented as mean ± SEM with statistical comparisons made using the unpaired student’s t-test and sourced in part from material published previously ^18^ under the Creative Commons licence.

TEER studies are carried out after a minimum of 6 weeks in culture as ARPE-19 cells do not form an effective barrier before this time ( Figure 7A–B). ARPE-19 cells also generate relatively poor barriers compared to hfRPE or PSC-RPE. However, the method describe herein can be adopted to test barriers created by RPE cells from different sources. Electrical recordings are obtained using an EVOM ^2^ epithelial voltohmmeter and a 4mm STX2 chopstick electrode (EVOM ^2^; 300523, World Precision Instruments Inc., USA). As importance is given to maintaining sterility in RPE cultured for long periods, electrodes are first sterilised in 70% ethanol, rinsed in ddH _2_O and equilibrated in pre-warmed culture medium prior to use. For this reason we also recommend performing a complete media change in both transwell compartments after measurements. Electrodes are inserted perpendicularly into the apical and basal compartments so that the tip of each arm is immersed in media. Five recordings are taken from each transwell at set time intervals (10 seconds) to calculate the average TEER value. Measurements are recorded from at least three separate transwell inserts. The reference value from a fibronectin coated transwell without cells is subtracted from initial measurements ( Equation 2) and the net recording corrected for area of cell growth to yield a final TEER value ( Equation 3, Table 2). All measurements are performed at room temperature within 6 minutes of removing cells from the incubator.

*Net TEER (Ω) = Measured TEER (Ω) - Reference TEER (Ω)*            [Equation 2]

*Final TEER (Ω/cm ^2^) = Net TEER (Ω) × Area of transwell membrane (cm ^2^)*            [Equation 3]

Technical tip: Care should be taken to prevent electrodes from touching chamber walls as this results in inconsistent TEER values.

**Step 8:** ELISA studies of ARPE-19 cultures

The capacity to secrete proteins directionally can be assessed by performing an ELISA on conditioned media harvested from apical and basal transwell compartments ( Figure 1). A Novex® human VEGF solid-phase sandwich ELISA kit (Life Technologies, UK) is used to measure secreted levels of VEGF, whilst a Biovendor Human PEDF solid-phase sandwich ELISA kit (Biovendor, UK) is used to measure secreted levels of PEDF. A complete media change is performed prior to quantifying soluble protein levels during a 2–3 day period. Collected samples are kept at 4°C or on ice before quantification to prevent protein degradation or stored at -80°C for future use. ELISA quantification is carried out in triplicate on a minimum of three separate wells ( Figure 7C–D). The volume of media lost due to sampling is restored afterwards by the addition of freshly prepared media into apical and basal transwell compartments. Assays are carried out following the manufacturers’ guidelines ( Table 6).

**Table 6.** ELISA kits used to detect VEGF and PEDF. Users should check assay detection thresholds and suitability for use with conditioned culture media.

Product Name	Supplier	Product Number	Technical Protocol/Guides
Novex ^®^ human Vascular Endothelial Growth Factor (VEGF) ELISA	Life Technologies, UK	KHG0111	https://www.thermofisher.com/order/catalog/product/KHG0111
Human Pigment Epithelium Derived Factor (PEDF) ELISA	BioVendor, Germany	RD191114200R	https://www.biovendor.com/pedf-human-elisa?d=114#undefined

**Step 9:** Photoreceptor outer segment phagocytosis assay

Post-confluent ARPE-19 cells are reported to exhibit phagocytic activity after 2 weeks in culture ^43^, although we recommend using cultures of approximately 4 months so cells exhibit a gene profile comparable to native RPE ^36^.

### Isolation of photoreceptor outer segments

Porcine eyes are obtained from a butcher or abattoir within 2 days of post mortem and POS isolated on the same day. An incision is made at the ora serata to remove the anterior eye portion after which the retina can be gently detached. We find this is best achieved by teasing the retina away from the RPE in a circular fashion. The optic nerve is severed at the nerve head to detach the retina. The retinae are pooled in KCl buffer (0.3M KCl, 10mM HEPES, 0.5mM CaCl2, 1mM MgCl2; pH 7.0) with 48% sucrose (w/v) and agitated vigorously for 2 minutes on a rotation mixer after which the solution is centrifuged for 5 minutes at 5,000g. At this point isolated POS appear in the supernatant and the pellet can be discarded. Filter the POS containing supernatant through a sterile surgical gauze positioned on a 1.5ml Eppendorf tube into an equal volume of KCl buffer without sucrose and incubate at room temperature for 5 minutes. Centrifuge the suspension at 4,000g for 7 minutes to pellet isolated POS and discard the supernatant. Wash POS pellets three times in 1xPBS and re-suspend in DMEM with 2.5% (w/v) sucrose ^44^. POS is covalently conjugated to fluorescein isothiocyanate (FITC). This is achieved by incubating pooled POS in 5ml labelling buffer (20Mm phosphate buffer pH 7.2, 5mM taurine with 10% w/v sucrose) and 1.5ml FITC stock solution (2mg/mL FITC isomer I in 0.1M Na2CO3 buffer; pH 9.5) on a rotator mixer at room temperature for 1 hour in the dark. Pellet the POS-FITC by centrifugation at 3,000g for 5 minutes, suspend in DMEM with 2.5% sucrose (w/v) and store for a maximum of 6 months at -80°C. Once thawed isolated POS should not be refrozen. The total protein content of POS preparations can be quantified using a BCA assay prior to use.

### Photoreceptor outer segment feeding assay

Cultures are incubated at 17°C for 30 minutes after which 4mg/cm ^2^ POS-FITC is applied to RPE cultures for 30 minutes to maximise binding with minimal internalisation ^39^. This concentration is sufficient to challenge each RPE cell with approximately 10 isolated POS molecules ^44^. Alternatively, if cultures cannot be chilled to 17°C they may be incubated with isolated POS for 2 hours at 37°C to achieve a similar effect ^45^. Following the feeding assay, wash inserts once in fresh medium and return to an incubator set at 37°C and 5% CO _2_. Transwells are removed at desired time points after which they are washed once in 1xHBSS followed by fixation in 1xPBS containing 4% formaldehyde for 30 minutes at 4°C. Wash cells three times in 1x PBS and store at 4°C until use. Immunostaining is performed by blocking/permeabilising cells in PBS-Tween containing 1% BSA for 30 minutes followed by incubation at 4°C overnight with the desired antibody ( Table 5) prepared in the same solution. The following day, wash cells three times with 1xPBS to remove any unbound primary antibodies and incubate with the appropriate secondary antibody (step 5) for 1 hour at room temperature. Wash samples as before and incubate with 1μg/ml DAPI (prepared in ddH _2_O) for 10 minutes before performing three final washes in 1xPBS. Mount the sample between two glass coverslips using Mowiol ^®^ mounting medium for confocal microscopy studies (step 5).

**Figure 8.**
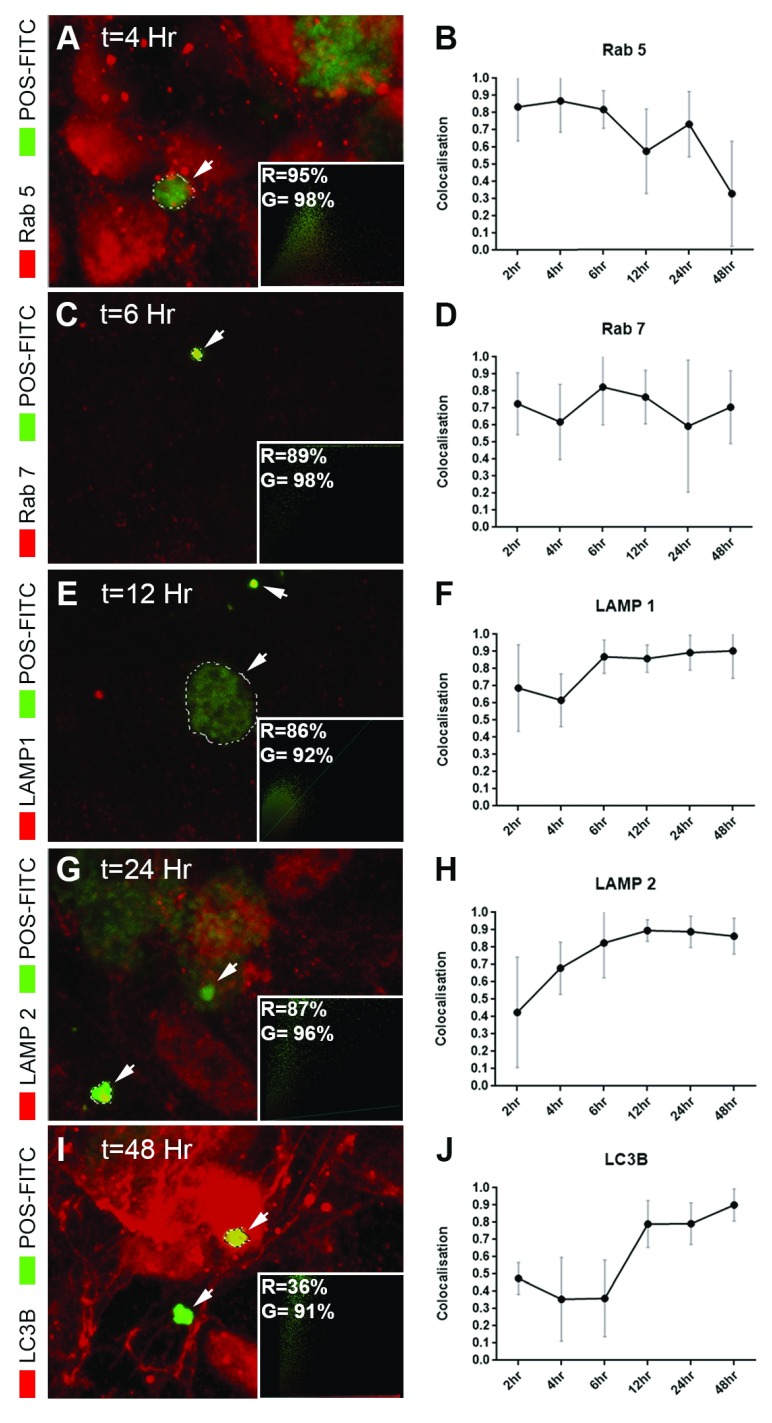
Intracellular trafficking and processing of photoreceptor outer segments in a feeding assay. Cultures were pulsed with photoreceptor outer segments (POS) bound to FITC. Cargo internalisation and trafficking via phagosomes/endosomes, lysosomes and autophagy bodies were quantified at 2, 4, 6, 12, 24 and 48 hours. Following internalisation, POS were detected [ **A**– **B**] in Rab 5 early vesicles by 2–4 hours. These diminished over time as cargo appeared [ **C**– **D**] in Rab 7-positive compartments. [ **E**– **F**] At 6 hours, a large proportion of POS were present in early lysosomes and [ **G**– **H**] in LAMP2 vesicles by 12–24 hours. [ **I**– **J**] Cargos appeared in LC3B-positive autophagy bodies afterwards which were present up to 48 hours. Images show representative confocal images with quantification (n=10 cells/compartment/time point) in 2 independent experiments. Inserts show extent of co-localisation in red (R) and green (G) channels using an unbiased quantification method by Volocity. Data is presented as mean ± SEM and sourced in part from material published previously ^18, 50^ under the Creative Commons licence.

For co-localisation studies we use an unbiased statistical algorithm described by Costes *et al.* ^40^ and performed using Volocity Software (Perkin Elmer, UK). Considerations prior to undertaking co-localisation studies include careful selection of suitable fluorophores to avoid bleed through and chromatic aberration as well as pixel saturation ( Figure 8). We also suggest selecting non-overlapping and non-adjacent fluorophores and refer to several excellent articles on co-localisation studies ^40, 46– 48^.

## Results

### Characterisation studies

ARPE-19 monolayers on transwell inserts can be easily maintained in long term culture ( Figure 1). This allows them to mature and express structural and physiological features of native RPE. Our experiments were carried out on monolayers that had been in culture for 2–4 months. We also tested the ability of ARPE-19 cells to attach and spread on transwell membranes with or without the presence of fibronectin; the preferred substrate for these cells in our experience ^18, 49, 50^. Our findings show that cells were capable of attachment and growth to confluence on PET membranes irrespective of the presence/absence of an underlying fibronectin matrix ( Figure 2). Prior to carrying out imaging studies transwell membranes were carefully removed from their plastic wells. We show a convenient method by which even a small transwell insert can be sectioned into several segments so that the investigator is able to probe for multiple markers and thus maximise the possibility of obtaining data from each transwell ( Figure 3). *In-vitro* RPE monolayers were studied for physiological and structural features characteristic of RPE cells. We first probed for junctional complexes zonula occludens (ZO-1) ( Figure 4A–D) and occludin ( Figure 4E–H). We also looked for evidence of apically expressed Na ^+/^K ^+ ^ATPase ( Figure 4I–L) and the cell-specific marker RPE65 ( Figure 4M–P). After 2 months in culture ARPE-19 monolayers expressed the early tight-junction protein ZO-1 with a border demarcating cell-to-cell contact, and 3D imaging revealing polarisation towards the apical cellular region. ZO-1 staining was also observed in the cytoplasm and prominently in the nucleus, which is consistent with reported literature ^51^. Expression of occludin during mid-late stages of barrier formation was also observed. Next, we probed for expression of the Na ^+^/K ^+^ ATPase transporter to assess the presence of a polarised plasma membrane. Na ^+/^K ^+^ ATPase is predominantly expressed on the apical RPE surface where it facilitates the process of photo-transduction. Apically expressed Na ^+^/K ^+^ ATPase is also linked with a highly differentiated, polarised RPE phenotype ^52^. We detected Na ^+^/K ^+^ ATPase in some but not all cells, although expression appeared to be limited to the apical RPE surface ( Figure 4L). We also probed for the cell-specific retinoid isomerohydrolase RPE65 marker to confirm identity of RPE cells in the monolayer ( Figure 4M–P). RPE65 was observed as punctate, cytoplasmic staining as reported by others ^53^ and confirmed the identity of RPE cells in long-term culture. Next, we assessed the extent to which ARPE-19 monolayers on transwells adopt ultrastructural features of native RPE. We describe a convenient technique by which transwell membranes can be sectioned into smaller segments to be embedded in resin blocks for TEM studies ( Figure 5). We observed evidence of numerous microvilli on the apical RPE surface ( Figure 6A) and infolded/convolutions of the basolateral cell membrane ( Figure 6B), characteristic of native RPE. Micrographs also showed details of intracellular organelles including mitochondria, compartments in the endocytic-lysosomal pathway and pigment molecules ( Figure 6C–E). Mitochondria, for instance, appear in cross-section as a double membrane-bound structures with luminal cristae, whilst vesicles contained cargos of varying electron densities. The arrangements of these organelles conformed to the apical-basolateral axis of native RPE. Junctional complexes between RPE, detected previously by immunofluorescence studies ( Figure 4A–H), were also observed at ultrastructural resolution as tight junctions and adherens junctions along membranes at the apical region of RPE cells ( Figure 6F–G). These were observed as electron-dense regions indicating points of cell-to-cell contact and associated with desmosomes in some instances.

### Validation studies

Establishment of an effective trans-epithelial barrier is a key feature of native RPE, and one that can be readily measured in transwell cultures. We carried out TEERs of ARPE-19 cultures over an approximately 3 month period. A stable electrical gradient was established following 6 weeks in culture ( Figure 7A), after which there were no appreciable changes to the barrier ( Figure 7B). An average TEER value of 40.72 Ω/cm ^2^ was noted once cultures had established a stable barrier in-line with previous reports ^22, 25^. Polarised secretion of molecules towards the overlying neuroretina and the underlying choroid is an important feature of RPE cells ^15^. Proteins such as VEGF and PEDF that are synthesised/secreted by RPE are known to possess pro-angiogenic and neuroprotective effects, respectively ^1^. Directional secretion of such molecules can easily be quantified in transwell compartments once cells establish an effective trans-epithelial barrier. To assess if this was achieved in culture we measured VEGF and PEDF levels in conditioned media using two different ELISAs. ARPE-19 cells secreted VEGF through both apical and basolateral surfaces at concentrations of 0.942 ± 0.035ng/ml and 2.852 ± 0.145ng/ml, respectively. VEGF secretion towards the choroid was therefore significantly higher compared to amounts released towards the neuroretina ( Figure 7C). PEDF levels were also secreted via both surfaces at concentrations of 16.95 ± 0.72ng/ml (apical) and 25.05 ± 3.93ng/ml (basal). Statistically, there were no differences in amounts of PEDF secreted towards the choroid or neuroretina ( Figure 7D). Next, we assessed the ability of cultured ARPE-19 cells to bind and internalise POS cargo. *In-situ* RPE daily internalises and proteolytically degrade POS from overlying photoreceptors, the impairment of which plays a key role in retinopathy ^1^. POS-FITC cargos were fed to 4 month old monolayers using a pulse-chase method described previously ^39^. Each compartment in the endosome/phagosome-lysosomal and autophagy pathway was assessed at 2, 4, 6, 12, 24 and 48 hours for the extent of co-localisation with fluorescently-labelled POS. Cargos initially appeared in early Rab 5 compartments ( Figure 8A–B), which by ~6 hours had trafficked to Rab 7 late vesicles ( Figure 8C–D). Between 6–12 hours, a large proportion of cargo had co-localised to early LAMP1 ( Figure 8E–F) and mature LAMP2A lysosomes ( Figure 8G–H). 48 hours after the pulse-chase assay was initiated, a large proportion of cargo appeared in LC3B-positive autophagy bodies ( Figure 8I–J).

Raw data underlying Figure 2 Click here for additional data file. Copyright: © 2018 Lynn SA et al. 2018 Data associated with the article are available under the terms of the Creative Commons Zero "No rights reserved" data waiver (CC0 1.0 Public domain dedication).

Raw data underlying Figure 4 and S1 Click here for additional data file. Copyright: © 2018 Lynn SA et al. 2018 Data associated with the article are available under the terms of the Creative Commons Zero "No rights reserved" data waiver (CC0 1.0 Public domain dedication).

Raw data underlying Figure 6 Click here for additional data file. Copyright: © 2018 Lynn SA et al. 2018 Data associated with the article are available under the terms of the Creative Commons Zero "No rights reserved" data waiver (CC0 1.0 Public domain dedication).

Raw data underlying Figure 7 Click here for additional data file. Copyright: © 2018 Lynn SA et al. 2018 Data associated with the article are available under the terms of the Creative Commons Zero "No rights reserved" data waiver (CC0 1.0 Public domain dedication).

Raw data underlying Figure 8 Click here for additional data file. Copyright: © 2018 Lynn SA et al. 2018 Data associated with the article are available under the terms of the Creative Commons Zero "No rights reserved" data waiver (CC0 1.0 Public domain dedication).

## Discussion

In this article, we describe a convenient protocol by which users can rapidly establish and study RPE cells in long-term culture. We used the human ARPE-19 cell-line, although the approaches described herein may be adopted for studying RPE from a variety of sources. We provide examples from our own laboratory as well as other groups to highlight the type of questions which investigators could realistically address using this model. These are by no means exhaustive as there is a considerable amount of literature that is beyond the scope of this article. Readers are directed to accompanying citations as well as to a special issue of Experimental Eye Research for detailed reviews covering specific aspects of RPE biology ^54^; Retinal Pigment Epithelium cell culture: Current standards and technical criteria for model systems (2014, Volume 126; 1–84, Edited by Bruce A. Pfeffer and Nancy J. Philp). We also discuss the versatility of transwell culture models as well as advantages and limitations of APRE-19 cells in particular, and where *in-vitro* models could replace similar work carried out in animals.

The use of transwell inserts to culture RPE is widely accepted as the best method to study this cell-type and to model dysfunction of this important tissue *in-vitro*. This has led to a plethora of studies in which RPE cells from different sources have been cultured on transwell systems ^16, 17, 19– 22, 24^. The use of transwell supports appear to mimic important structural features of the BrM, as cells displayed desirable structural and functional features of native RPE. Limitations to this approach are driven largely by the source/type of RPE cells as well as differences in culture conditions. For instance, the culture of hfRPE on transwell inserts produced what some consider to be the holy grail of RPE culture by mimicking drusen formation associated with complement activation ^55^. Further advances in RPE modelling were made by culturing PSC-RPE from Sorsby fundus dystrophy, Doyne honeycomb retinal dystrophy/malattia Leventinese and autosomal dominant radial drusen patients, which recapitulated important RPE-associated disease features *in-vitro* ^56^. Use of the ARPE-19 cell-line by contrast, which had been in use for significantly longer, appeared to be less attractive. When cultured under certain growth conditions these cells failed to replicate directional secretion of proteins, showed limited evidence of pigmentation and impaired retinoid metabolism as well as poor expression of markers PMEL17, BEST1, CRALBP and MerTK ^22, 25, 57^. More recently however, it has been demonstrated that when cultured under optimised growth medium on transwell inserts for extended periods, ARPE-19 cells regain a phenotype and gene expression profile comparable to that of native RPE cells ^25, 36^. Moreover, recent improvements to ARPE-19 culture meant that features that were difficult to reproduce previously including directional secretion of proteins, apical-basolateral morphology, pigmentation, internalisation of POS and expression of mRNA/proteins in retinoid metabolism have all been successfully recapitulated ^18, 25^. In fact, when cultured for ~4 months, the genetic profile of ARPE-19 cells were found to be comparable to hfRPE and PSC-RPE ^36^. Through the adoption of high glucose and sodium pyruvate media described by Ahmado and colleagues ^25^ as well as other advances described herein, we and others have collectively improved the capacity to exploit ARPE-19 cells to model specific aspects of retinopathy under culture conditions. For example, we have shown that after two months in culture, VEGF and PEDF are secreted by ARPE-19 at concentrations similar to levels reported in hfRPE ^18, 22^. *In-vitro* models such as those described herein provide a convenient method to assay directional secretion of molecules which would otherwise be challenging to study using *in-situ* RPE in mouse models. We also show the presence of desirable ultrastructural features after 2 months in culture including apical-basolateral specialisation and presence of apically distributed pigment which typically become visible without a microscope after 3–4 months in culture ^18, 25^. Similarly, for the first time, we report the presence of RPE65 and the apical expression of Na ^+^/K ^+^ ATPase after just 2 months in culture ^18^. Na ^+^/K ^+^ ATPase expression however was limited to a sub-population of RPE cells. A similar observation was made by others after 15 weeks in culture ^25^, suggesting a potential limitation of this cell-line. Given that the expression of this transporter is correlated with the increased pigment ^25^, we recommend maintaining ARPE-19 cultures for 2–4 months before carrying out any studies on trans-epithelial transport.

ARPE-19 cells express the appropriate surface receptors and ligands for binding to POS. Investigators have therefore exploited these cells to study cargo trafficking and impairment of this process which is associated with disease ^50, 58^. However, some caution is advised as post confluent ARPE-19 cells were reported to internalise cargo after only 2 weeks without expressing MerTK (for POS internalisation) or ITGAV (for POS recognition/binding) receptors until at least 4 months in culture ^36^. We therefore recommend that POS binding and trafficking studies should only be carried out after this period. Investigators should also note that cargo trafficking rates differ somewhat between primary RPE and ARPE-19 cells with slower speeds reported in the latter ^58^. Using a pulse-chase assay we used cultures to obtain a detailed timeline of POS trafficking in ARPE-19 cells ^18, 50^. Our findings reveal the time wise entry of POS to Rab 5-positive early compartments followed by Rab 7 late vesicles and appearance in lysosomes between 6–12 hours. Cargos were detected in LC3B autophagy bodies as late as 48 hours after pulse chase, although a majority of cargo were degraded within 16–20 hours as reported before ^58^. Importantly, such new information defining trafficking rates in heathy cells may be used as a reference point in modelling specific disease conditions, and how they affects POS processing and degradation linked to retinopathies. These studies are far easier to manipulate and carryout under *in-vitro* conditions hence investigators may prefer to eschew mouse models for this type of work.

Another important feature of the RPE modelled under *in-vitro* conditions is their capacity to develop and form junctional complexes integral to creating the BRB ^14^. Although we observed the expression of early and mid-late barrier complexes after 2 months in culture by confocal microscopy as well as tight junctions and adherens junctions at ultrastructural resolution, ARPE-19 cells appear to be less well suited to barrier studies. ZO-1 is reported to shuttle to/from the nucleus depending on the extent and maturity of tight junctions ^51, 59^. We observed strong ZO-1 staining in cell margins as well as in nuclei after 8 weeks, suggesting a potentially incomplete maturation of the barrier. Staining for occludin was also limited to circumferential tight junctions. 15 week old ARPE-19 cells however displayed a stronger pattern of staining for ZO-1 and occludin ^25^, suggesting a degree of on-going barrier maturation at earlier time points (8 weeks) at which our experiments were carried out. Immature and/or incomplete barrier complexes appear to be reflected in low TEER values for ARPE-19 cells, which is seldom reported to exceed 50 Ω/cm ^2^ ^25^. We recorded an average TEER of 40.7 Ω/cm ^2^ over a 3 month period, which was substantially less compared to values of 200–1500 Ω/cm ^2^ reported in RPE cells from other sources ^33^. For these reasons we do not advocate the use of the ARPE-19 cell-line for barrier studies.

In summary, this article provides an in-depth set-up and validation protocol for establishing a culture model of the outer retina using the widely utilized ARPE-19 cell-line. We also discussed advantages and limitations of transwell models in general and ARPE-19 cells in particular, so that users may best exploit this versatile system for their studies. Advantages over mouse models such as (1) its use as a viable alternative, (2) ability to rapidly generate functional RPE monolayers akin to native tissues, and (3) ability to reproduce disease features that could only be previously studied in mice ^18, 55, 56^, makes *in-vitro* models of the outer retina especially attractive. Their versatility is further demonstrated by studies in which the surface of transwell membranes are directly modified to mimic effects of aging ^23^. Investigators are also well-placed to take advantage of new developments in stem-cell technology and refinements to *in-vitro* cultures described herein ^60– 62^ as well as a plethora of artificial BrM substrates on offer. The latter may be set-up and assembled similarly to transwells by using commercially available products such as CellCrown ^TM^ inserts (Sigma, UK). The growing interest in microfluidic devices allow laboratories to model relationships between the RPE vs. choroidal endothelial cells and blood flow by incorporating the latter into transwell devices. These advances combined with the development of fast/high-resolution imaging and new 3D imaging platforms such as serial block face scanning electron microscopy and Lightsheet are likely to usher in further opportunities to exploit *in-vitro* models. Consequently, investigators may wish to consider these culture models as attractive alternatives to using animals, or at least as powerful new tools to be exploited in parallel that will also have the benefit of reducing and replacing animals used in research. 

## Data availability

The data referenced by this article are under copyright with the following copyright statement: Copyright: © 2018 Lynn SA et al.

Data associated with the article are available under the terms of the Creative Commons Zero "No rights reserved" data waiver (CC0 1.0 Public domain dedication). 

Dataset 1: Raw data underlying Figure 2 10.5256/f1000research.15409.d209252 ^63^

Dataset 2: Raw data underlying Figure 4 and S1 10.5256/f1000research.15409.d209253 ^64^

Dataset 3: Raw data underlying Figure 6 10.5256/f1000research.15409.d209254 ^65^

Dataset 4: Raw data underlying Figure 7 10.5256/f1000research.15409.d209255 ^66^

Dataset 5: Raw data underlying Figure 8 10.5256/f1000research.15409.d209256 ^67^

Figure 4, Figure 6, Figure 7 and Figure 8 has been published previously either in part or in whole (Ratnayaka *et al*., IOVS 2015 ^49^; Lynn *et al*., 2017: doi: 10.1016/j.tice.2017.06.003 ^18^; Keeling *et al*., 2018: doi: 10.3390/cells7020016 ^50^), and presented here under the terms of the Creative Commons (CC) license.

## Supplementary material

**Figure S1**: Secondary antibody controls used to detect Retinal Pigment Epithelium proteins in ARPE-19 cultures on transwells. [ **A**– **C**] Probing with goat anti-rabbit Alexa 594 (red) or [ **D**– **F**] goat anti-mouse Alexa 594 (red) produced no signals. Nuclei were labelled with DAPI (blue). Scale bars correspond to 20 µm.
