# Supplementary figures and images for: A convenient protocol for establishing a human cell culture model of the outer retina
Source: F1000Res. 2018 Jul 18;7:1107. [Version 1] doi: 10.12688/f1000research.15409.1 (PMC6137423; doi:10.12688/f1000research.15409.1)

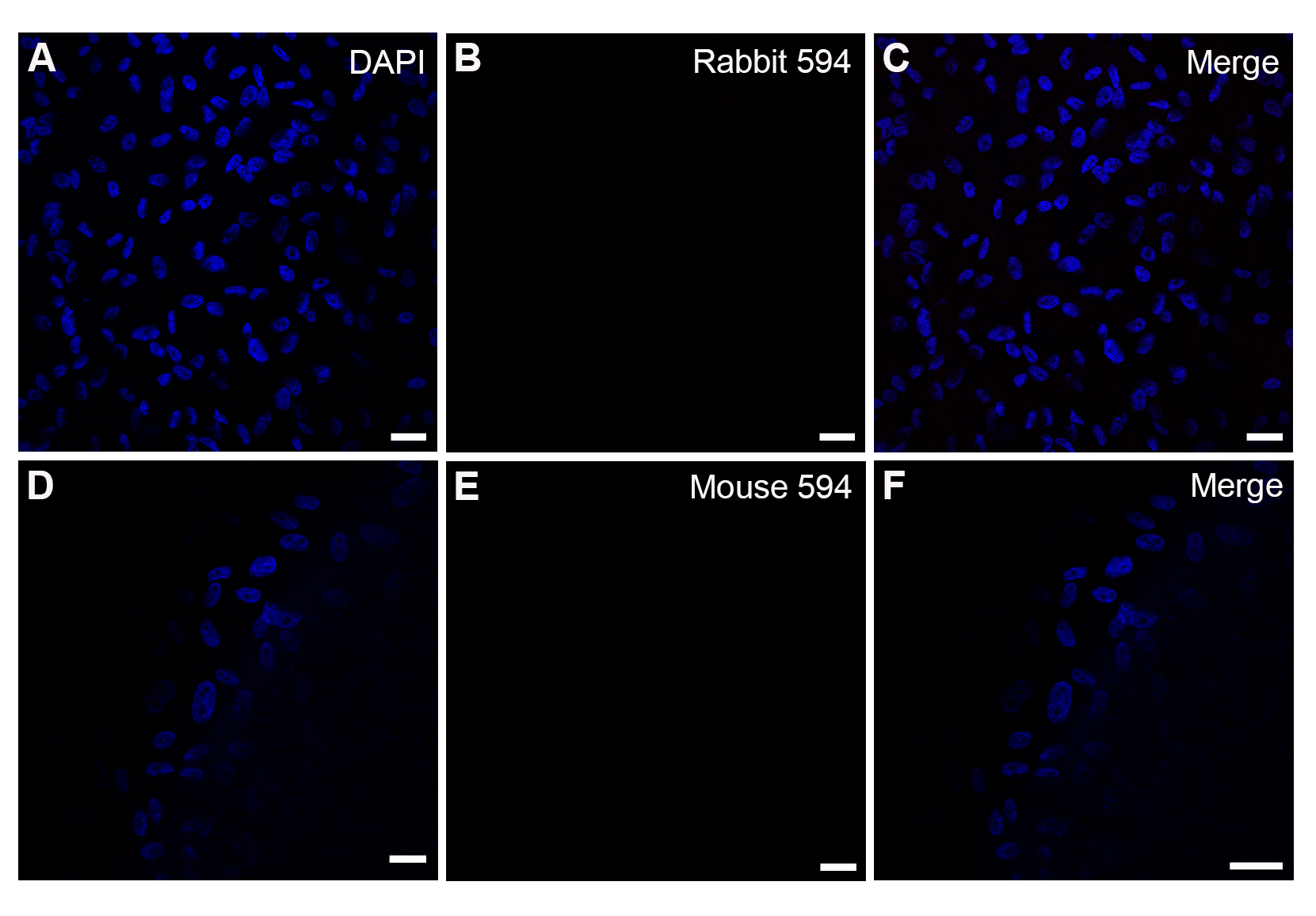

Supplement: Supplementary file 6 [file f1000research-7-16791-s0005.tgz › 5f2bdb5a-02e9-408e-a691-85f67978714c.png]
