# Supplementary material for: A convenient protocol for establishing a human cell culture model of the outer retina
Source: F1000Res. 2018 Jul 18;7:1107. [Version 1] doi: 10.12688/f1000research.15409.1 (PMC6137423; doi:10.12688/f1000research.15409.1)
Supplement: Peer review history

# Peer review: Referee response for version 1

**DOI:** 10.5256/f1000research.16791.r37636

**Published:** 2018-09-13

**Authors:** Ian J. Jackson

**Affiliations:** 1 MRC Human Genetics Unit, Institute of Genetics and Molecular Medicine, University of Edinburgh, Edinburgh, UK

This paper is describing the culture of ARPE-19 cells on fibronectin-coated membranes. Cultures of ARPE-19 cells have been used for some time as models of retinal pigment epithelium. Other cell types are used: human fetal RPE cells, pig or mouse primary RPE cells and latterly human induced pluripotent stem cells and embryonic stem cells. The method described overall is not novel, but it is very well documented in considerable detail in this paper. The use of ARPE-19 cells has some merits in terms of reproducibility.

 Whilst differentiated iPS cells from patients is a means of investigating diseases processes in vitro, this is time consuming and technically challenging. On the other hand ARPE-19 cells have been edited by CRISPR and could be used to the same ends possibly more easily. Further engineering of ARPE-19 cells could introduce, for example, reporters for live imaging.

 The methodology is clear and the protocols are detailed.

 The results as presented are clear. Once downloaded and unzipped, the source data are clear

 In the Abstract the authors say that they are describing a model of the outer retina incorporating the RPE and the supportive Bruch's membrane. I think it is debatable whether this is a good model of Bruch's membrane; there isn't a full barrier established, for example. In their characterisation they compare their data to previously published data from other systems; this isn't ideal as there could be protocol and lab differences. It would also be worth noting in the Discussion that previous work (Ablonczy et al 2011, their ref 22) has compared ARPE-19 cells to human fetal RPE cells grown on permeable filters and concluded that hfRPE cells were a better model. They concluded that ARPE-19 cells resembled a pathological RPE.

**Are a suitable application and appropriate end-users identified?**

 Yes

**If applicable, is the statistical analysis and its interpretation appropriate?**

 I cannot comment. A qualified statistician is required.

**Are the 3Rs implications of the work described accurately?**

 Yes

I have read this submission. I believe that I have an appropriate level of expertise to confirm that it is of an acceptable scientific standard.

# Peer review: Referee response for version 1

**DOI:** 10.5256/f1000research.16791.r36346

**Published:** 2018-08-14

**Authors:** David Y.S. Chau

**Affiliations:** 1 Research Centre inTopical Drug Delivery and Toxicology, Department of Pharmacy, Pharmacology and Postgraduate Medicine, School of Life and Medical Sciences, University of Hertfordshire, Hatfield, UK

**Overview:**

 This article aims to address the utilisation of a viable, *in vitro*, retinal pigment epithelium (RPE) cell culture model, as an alternative to animal models, for disease elucidation and the ageing mechanism. Specifically, the article describes a novel, step-by-step, technique to establish an experimentally versatile model of the outer retina- incorporating the RPE monolayer and associated Bruch’s membrane (BrM).

**General Assessment:**

*Introduction: *Concise overview and summary of RPE cells and aligns the importance of this layer and to disease aetiology. Examples of current *in vivo* models used for the assessment of retinal pathobiology is mentioned and the exploitation of mice is emphasised appropriately- although the inclusion of absolute specific numbers of rodents/mice should be stated if possible. For completeness of 3R, a sentence regarding *in silico*/computational alternate retinal models should be included in the Introduction. The authors correctly highlight the importance of a suitable microenvironment to the induction/differentiation of the (RPE) cells and emphasis is placed on Transwells. However, a brief mention of additional stimuli/factors should also be elaborated upon including use of hydrogels, co-culture, surface topography, surface geometry/curvature, growth factors- see literature. Good overview of ARPE-19 cells- with emphasis of it being human cell line. Should a critique of the use of human donor tissue/cells be mentioned- from a 3R perspective?

*M&M: *It may be worth stating the original passage number of these cells, if known and/or since purchase, as the culture methodology is dependent on low/high status c.f. Dunn *et al*., 1996, Ahmado *et al.*, 2011, Samuel *et al., *2017. Would using 1% human serum and/or serum-alternative be possible- considering the implication of the 3R? Why fibronectin coating and not the other ECM proteins/laminin/Matrigel- laminin is a component of basal RPE lamina? The coating exploits surface adsorption- why do you let the Transwell “dry” overnight and how can you aspirate residual fibronectin if so- rather to “coat” overnight? Confirmation of the presence of fibronectin coating was achieved by? Agitation during the coating procedure? Is the ELISA a single timepoint at 2-months or cumulative collection of media- how is this achieved when there are frequent media changes required? FBS contains exogenous (ECM) proteins as well as VEGF and PEDF? Please state “room temperature” for TEER measurement (and POS)- I assume that this was performed in a hood rather than “on the bench” hence airflow/room temperature appropriate? Capital “T” for Transwell throughout.- registered product.

*Protocol:*

**Step 1**: Additionally filter step may be seen as “overkill”- an additional step could actually increase infection risk- reagents can simply be added using aseptic technique? Please mention storage condition and “warming-up” protocol for media. Discrepancy to name of “FBS” mentioned in the M&M and Table 1: FBS = foetal calf serum; N4762 is newborn calf serum (NBCS)- difference in composition especially antibodies and exogenous protein profile.

**Step 2:** Amend to ensure that the FN-coating step is mentioned before the seeding of cells- the former is required before the latter and should also tally with Figure 1. What % confluency is reached before media change? The 6-minute trypsin protocol is very exact- did you confirm visually under a microscope? Over/under-incubating can be problematic as it may lead to selective population isolation- also, as the author stated, longer-term cells generate their own ECM which will enhance the cells’ adhesiveness to the substrate. Was a simple viability and cell count i.e. trypan blue performed- needs to be mentioned in Step 4- determine initial seeding densities?

**Step 3:** any agitation used during the coating process?

**Step 3 (technical tip): **fibronectin solution should never be vortexed/centrifuged either- also results in “crashing out” of solution. Note that “steps” in Figure 1 and main text are out of synch- please amend e.g. Step 3 in Figure 1 is “seeding” whereas in the text, Step 4 is seeding the Transwell.

**Step 4**: State volume applied to apical and basal compartments prior to seeding? What volume was the cell suspension when adding to the Transwells- possible sink conditions? Clarify what “obvious signs of pigmentation is”.

**Step 4 (technical tip)**: was there a slight agitation when cells were first added to ensure uniform coating of substrate?

**Step 5: **Steps out of sync with Figure legend. Can anything about morphology be discussed from the images? The study emphasises the long-term ability of the culture conditions i.e. 2-4 months- do you have any associated images for the extended timepoints to indicate viability and/or correct phenotype? Previous researchers have exploited wax embedding as a way to preserve/protect the samples before imaging- similar to your TEM preparation- any reason/justification/mention of the suitability? Capital A in antibodies. What confocal was used and associated operating conditions required. Could Figure 4, panel I, J and K be recomposed with the same magnification/scale as the other panels- allow easier comparison. State timepoint of image acquisition- assuming 2 months based on RPE65 sentence? Full name for NGS required- normal goat serum?

**Step 6: **State the dimensions/manufacturer of the embedding capsule used. Details for Reynold’s Lead stain required i.e. citrate? concentration, time.

**Isolation of POS:** Please state pore size/manufacturer for the surgical gauze used for filtration.

**POS feeding assay: **how were the samples chilled/incubated to 17°C- state instrument used and/or humidified/gaseous incubator? Optimal internalisation occurs at 37°C- so why 2 hours at 37°C same as 30 minutes at 17°C?

*Results: *Authors comment on attachment and spreading characteristics but have not alluded to what is ideal/typical of RPE cells- expansion needed regarding morphology of cells. Growth, per se, has not really been documented- absolute cell count, mitochondrial activity assessment i.e. MTT/XTT/MTS assays required alongside a complementary LDH-release profile would fully confirm that the cells are active, alive and not subject to (induced) death i.e. apoptosis or necrosis. No cell numbers are mentioned throughout the long-term culture aside from the initial cell seeding density and justification of contact inhibition. Appropriate representative markers have been selected and results suggest that the cells are of the correct phenotype, barrier formation and correct functionality. 

*Discussion: *A good descriptive and critical overview is provided by the authors. However, there is actually no direct incorporation of a Bruch’s membrane e.g. ex vivo tissue or biomimetic collagen-elastin substitute within this study which contrasts the statement(s) made in the Abstract and Research Highlights. The authors also point out the shortfall of their model e.g. ATPase expression, TEER/barrier function, POS binding. I would like a bit more discussion with regards to how this technique/protocol could be applied to mimic diseased states- is it simply culturing cells from (diseased) donor tissues/cells? Would a full (re)validation of culture conditions be required or is the assumption that Transwell plus culture media optimisation would suffice? Could this be incorporated within a 3D model? Multilayer/co-culture format? Slight expansion on future integration in ocular toxicity assessment and drug discovery would be beneficial.

*References: *All appropriate- suitable and up-to-date

*Supplementary material: *All appropriate and relevant

**Are a suitable application and appropriate end-users identified?**

 Yes

**If applicable, is the statistical analysis and its interpretation appropriate?**

 Yes

**Are the 3Rs implications of the work described accurately?**

 Yes

I have read this submission. I believe that I have an appropriate level of expertise to confirm that it is of an acceptable scientific standard.
